# Pathways explaining racial/ethnic and socio-economic disparities in incident all-cause dementia among older US adults across income groups

**DOI:** 10.1038/s41398-022-02243-y

**Published:** 2022-11-15

**Authors:** May A. Beydoun, Hind A. Beydoun, Sri Banerjee, Jordan Weiss, Michele K. Evans, Alan B. Zonderman

**Affiliations:** 1 Laboratory of Epidemiology and Population Sciences, National Institutes on Aging, NIA/NIH/IRP, Baltimore, MD USA; 2 Department of Research Programs, Fort Belvoir Community Hospital, Fort Belvoir, VA US; 3 College of Health Professions, School of Health Sciences, Walden University, Baltimore, MD US; 4 Stanford Center on Longevity, Stanford University, Stanford, CA US

**Keywords:** Predictive markers, Human behaviour, Long-term memory

## Abstract

Differential racial and socioeconomic disparities in dementia incidence across income groups and their underlying mechanisms remain largely unknown. A retrospective cohort study examining all-cause dementia incidence across income groups was conducted linking third National Health and Nutrition Examination Surveys (NHANES III) to Centers for Medicare and Medicaid Services-Medicare data over ≤26 y of follow-up (1988–2014). Cox regression and generalized structural equations models (GSEM) were constructed among adults aged≥60 y at baseline (*N* = 4,592). Non-Hispanic Black versus White (NHW) adults had higher risk of dementia in age and sex-adjusted Cox regression models (HR = 1.34, 95%CI: 1.15–1.55, *P* < 0.001), an association that was attenuated in the SES-adjusted model (HR = 1.15, 95%CI: 1.01–1.34, *P* = 0.092). SES was inversely related to dementia risk overall (per Standard Deviation, HR = 0.80, 95% CI:0.69–0.92, *P* = 0.002, Model 2), mainly within the middle-income group. Within the lowest and middle-income groups and in socio-economic status (SES)-adjusted models, Mexican American participants were at lower all-cause dementia risk compared with their NHW counterparts. GSEM models further detected 3 pathways explaining >55% of the total effect of SES on dementia risk (Total effect = −0.160 ± 0.067, *p* = 0.022), namely SES→LIFESTYLE→DEMENTIA (Indirect effect (IE) = −0.041 ± 0.014, *p* = 0.004), SES→LIFESTYLE→COGN→DEMENTIA (IE = −0.006 ± 0.001, *p* < 0.001), SES→COGN→DEMENTIA(IE = −0.040 ± 0.008, *p* < 0.001), with the last two remaining significant or marginally significant in the uppermost income groups. Diet and social support were among key lifestyle factors involved in socio-economic disparities in dementia incidence. We provide evidence for modifiable risk factors that may delay dementia onset differentially across poverty-income ratio groups, underscoring their importance for future observational and intervention studies.

## Introduction

Cognitive function refers to those mental processes that are crucial for the conduct of the activities of daily living, while dementia is defined as impaired abilities related to memory, thought processes and decision-making that is not part of normal aging which interferes with doing those activities of daily living [1]. Alzheimer’s Disease (AD) is the most common form of dementia and is currently the 7^th^ leading cause of death in the United States [2]. An estimated 6.5 million US adults over the age of 65 years are living with AD, a figure predicted to reach 13.8 million by 2060 [2]. Currently, the global dementia prevalence is estimated at ~4.7% [1], with the myriad of social determinants identified as being related to both dementia and cognitive function, including low income, poor education and racial minority status [3, 4]. Dementia onset has been previously shown to occur at earlier times in minority groups compared to NHW, with potentially greater survival among minority groups with AD [5–8]. In addition, dementia onset was also shown to be earlier within lower socio-economic status groups (SES, measured with income and education) [8–15].

Multiple pathways have been proposed in relating low SES or racial minority status to cognitive health, including differentials in lifestyle and behavioral characteristics (e.g. diet, physical activity, smoking, and social engagement) across race and income [3, 4, 16–23]. Disparities in cognitive health also parallel differences in education status [4]. Chronic stress, assessed through the accumulation of allostatic load [24], is another mechanism by which lower SES or racial minority status may contribute to cognitive health [3, 25]. Brain markers of cognitive aging, including the volume of the prefrontal cortex have been shown to be negatively affected by chronic stress, a byproduct of low SES [26]. Significant positive effects of income on brain surface area, volumes and white matter integrity in numerous regions of interest were detected in previous studies, including those predictive of better cognition across the life span [27–29].

Overriding brain pathology suggestive of AD dementia, some individuals present with no symptoms [4]. In fact, approximately two-thirds of cognitively normal subjects assessed longitudinally meet pathological criteria for AD at autopsy [30, 31]. Individuals with high educational attainment and higher reserve capacity may compensate for the damage arising from age or disease-related neurodegeneration [4]. This is demonstrated by better-than-expected cognitive performance and, thereby better coping with dementia onset through maintenance of normal functioning longer than individuals with lower educational attainment [4].

The underlying mechanisms by which racial minority status and lower SES may be related to dementia risk have not been systematically explored, particularly across income groups. We used data on older adults from the third National Health and Nutrition Survey (NHANES), which has undergone linkage with the Centers for Medicare and Medicaid Services (CMS)-Medicare follow-up data, in order to test racial/ethnic and socioeconomic disparities in dementia incidence across income groups, further exploring pathways through lifestyle, health-related and cognition-related factors.

## Materials and methods

### Database

The NHANES is a series of cross-sectional surveys sponsored by the National Center for Health Statistics (NCHS). It provides nationally representative data focused on population health and nutritional status in the United States using a stratified, multistage probability cluster design sampling methodology. Data collection involved in-home interviews on basic health and demographics followed by in-depth examinations in mobile examination centers (MEC) [32]. Details of CMS-Medicare and National Death Index (NDI) linkage methodologies are provided in Appendix I. The Institutional Review Board of the National Institute on Aging, Intramural Research Program approved this study with ethical treatment of participants.

### Study sample

Figure S1 shows the participant selection flowchart. We selected all NHANES III (1988–1994) participants aged ≥60 y having complete data on cognitive performance tests, CMS-Medicare data, with HMO exclusion. Of the initial 33,199 participants (aged 1–90 y), the final sample consisted of 4,592 participants, with no further exclusions applied due to multiple imputation (% missing <10% beyond cognitive performance test exclusion).

### Incident dementia

Dementia incidence was defined using information from the CMS Chronic Condition Data Warehouse Categories containing 21 chronic conditions (varying reference time periods), numbers and types of claims to qualify, exclusions and a set of ICD-9/CPT4/HCPCS codes. ICD-9 codes 331.0 for AD or several other ICD-9 codes listed in Appendix I for non-AD dementia (any claim DX), from inpatient, Skilled Nursing Facilities (SNF), home health agencies (HHA), Health Options Program (HOP) or Carrier claims over a 3-year period. Age, the underlying time scale, was calculated with exact dates starting from Medical Examination Center (MEC) examination age. Follow-up was from 1999 through end of 2013 for predetermined earliest occurrence date. The same algorithm to estimate dementia’s earliest diagnosis date was used for the 1991–1998 follow-up period [33]. AD mortality was incorporated into the outcome when incidence of AD was not directly available from Medicare data. However, mortality in general was incorporated into the outcome to censor the follow-up time upon death of the participant, and the last follow-up date was set at December 31^st^, 2014 for individuals who survived based on NDI data.

### Exposure

The main study exposures were racial/ethnic status (RACE_ETHN), with Non-Hispanic White (NHW), selected as the reference category (i.e. Non-Hispanic Black [NHB] vs. NHW, Mexican-American [MA] vs. NHW, OTHER vs. NHW, Non-White vs. NHW). Race/ethnicity and sex were self-identified.

### Mediators and moderators

#### Socioeconomic status

Socioeconomic status (SES) was defined by combining continuous poverty income ratio (PIR) and education (years) into a single z-score, while taking the average of education and PIR z-score, after a principal components analysis (PCA) was conducted. In addition, PIR was categorized in to “lowest income group” with a cutoff of 130%, while the middle-income group was between >130% and ≤300%, and finally the “highest income group” had a PIR > 300%. This categorical income measurement was the main moderator in our analyses. The 130% cutoff was chosen in other studies, given that in many States, individuals and families within that group would be eligible for federal or State benefits. In addition, 130% roughly represents the lowest income quintile in this wave of NHANES, especially among older adults. The 130–300% represents the income group remaining ineligible for benefits, despite their economic constraints. Finally, the group that is 300% PIR or higher, is considered to have a livable income, and constitutes the uppermost two quintiles of PIR in this age group. In the overall group of older adults (aged 60 + y), the lowest chosen income group corresponded to a mean ± SD educational attainment of 6.9 ± 4.1, while the middle-income category corresponded to 9.7 ± 3.7 years, and the uppermost income category to 12.6 ± 3.2 years. In addition, when SES PCA score was computed within each income group, % of variance explained by comparable across income groups, with the first principal component explaining 60–65% of the total variance of education and PIR within each income category.

#### Lifestyle and social support factors

Factors included the “SMOKING”, “ALCOHOL”, “DIET”, “NUTR”, “PA”, and “SS” constructs (Appendix II). Their operationalization was similar to SES, whereby positively correlated measured variables within each construct, based on results of PCA, were combined taking the mean of their respective standardized z-scores. A single 24-hr dietary recall was available for NHANES III, with interviews conducted by bilingual-trained MEC staff and data collected using computer-automated, interactive Dietary Data Collection system. Measuring guides aided portion size estimation, while dietary data were coded using seven-digit food codes from the US Department of Agriculture survey nutrient database [34, 35]. Upon estimation of nutrient intakes with a database provided for NHANES III [36], two measures of diet quality were derived, namely the 1995-Healthy Eating Index (1995-HEI) ranging from 0–100 [37] and the mean adequacy ratio score (MAR), [38–40]. “DIET” is the average of z-scores of those two total scores, similar to “SES”, while “NUTR” combined serum folate, vitamin A, vitamin E and total carotenoids. Alcohol use (grams/day) consisted of a single item from the 24-hr dietary recall, which was z-scored (ALCOHOL). “PA” (physical activity) combined 3 items comparing activity to past year, age peers and self, 10 years ago, respectively; SMOKING was measured with two items, reflecting number and years of cigarettes smoked; “Social support” (SS) with 5 items, combined into one average z-score, reflecting frequency (# per year) of five types of contacts (1) telephone with family, friends, or neighbors, (2) Getting together with friends or relatives, (3) visit with neighbors, (4) Attending church or religious services, and (5) Attending meetings at clubs or organizations.

#### Poor Health construct

“HEALTH” was operationalized with 4 ordinal or continuous items (average of their z-scores), namely self-rated health, co-morbidity index, body mass index (BMI, weight/height-squared, kg/m^2^) and the allostatic load (AL) score (range: 0–9, Appendix II), [41] coded as poorer health having higher scores (average of 4 *z*-scores). Participants rated their own health as: “Excellent” (referent), “Very good”, “Good”, “Fair” or “Poor”, while the co-morbidity index resulted from the sum of 14 possible self-reported conditions, namely “arthritis“, “congestive heart failure“, “stroke“, “asthma“, “chronic bronchitis“, “emphysema“, “hay fever“, “cataracts“, “goiter“, “thyroid disease“, “lupus“, “gout“, “skin cancer”, “other cancer”.

#### Cognitive performance tests and Poor cognition (COGN) summary PCA score

A battery of cognitive performance test scores was available in an NHANES III sub-sample aged ≥60 y. Four test scores were combined into a summary PCA score that reflects poorer performance with higher score (COGN). The four test scores used to define COGN were derived from Word recall, Story recall, Math/arithmetic (Serial 3’s) tests (Appendix III**)**.

### Exogenous covariates

Exogenous covariates were defined as variables that predict mediators and final outcomes in all models. In addition to age and sex, those consisted of, marital status (1 = Never married, 2 = Married, 3 = Divorced, 4 = Widowed, 5 = Other), household size and urban-rural residence (1 = Urban, 2 = Rural).

### Statistical methods

Using Stata 16.0 (StataCorp, College Station, TX) [42], we accounted for sampling design complexity [43] by incorporating appropriate sampling weights, primary sampling units and strata. Aside from cognitive performance, which determined the largest sample size, race/ethnicity (and other socio-demographics) or incident dementia outcome, which were largely complete, covariates were multivariate-imputed with chained equations [44, 45]. Consequently, population estimates of means, proportions and regression coefficients were obtained with Stata survey (svy) commands, computing standard errors (SE) with Taylor series linearization [43]. Comparison across income groups were made using svy:reg and svy:mlogit commands, with each of “medium” and “highest” income groups contrasted with “lowest” income group.

Age-to-event (in years) was defined from age at entry≥60 y (i.e. delayed entry) until exit age when event of interest has occurred or through censoring (death or end of follow-up). We estimated nested and income-stratified Cox proportional hazards (PH) models for all-cause dementia outcome, using multiple-imputed data. Socio-demographic, SES, lifestyle (diet, nutritional biomarkers, smoking, alcohol, social support, and physical activity), health, and cognitive performance factors were entered consecutively in five models. Heterogeneity of race and SES effects by income group was also tested. Mediating effects of each of these factors were examined using discrete-time survival analysis within the GSEM framework, also accounting for sampling design complexity within imputed data, a method deemed optimal when testing causal mediation in survival analysis. [46] Within GSEM, Logit(Hazard_dementia_) was the final dependent variable in person-period data, namely 5-year periods from age at entry till exit, in a model that included ≤6 age periods per person (65–70 y till 85 + y (referent category)). Exogenous socio-demographic variables were adjusted for in all equations. These variables in addition to RACE_ETHN (Non-White vs. White), included age, sex, marital status, household size and urban/rural area of residence. This analysis was conducted in each income group separately and results were compared qualitatively. The GSEM models allowed SES z-score to predict six constructs (“ALCOHOL”, “DIET”, “NUTR”, “PA”, “SMOKING” and “SS”), which then predicted the “HEALTH” standardized *z*-score. HEALTH subsequently was tested as a predictor for “COGN” (higher z-score → poorer performance), which was hypothesized to directly affect DEMENTIA risk. Importantly, other pathways allowed included those between endogenous variables (e.g. SES→HEALTH; SES→COGN; LIFESTYLE→COGN; LIFESTYLE→DEMENTIA; HEALTH→DEMENTIA) and between RACE_ETHN and each endogenous variable (Fig. S2). Furthermore, selected indirect effects of RACE_ETHN and SES on dementia risk were examined across income groups, while indirect effects of SES on dementia risk were also examined in the overall population.

GSEM was conducted averaging results across 5 imputations, using Rubin’s rule [47]. From these results, direct effects are presented in a structured manner to represent the main pathway, direct effects into the final DEMENTIA outcome, relationships between endogenous variables outside the main pathway, and direct effects of RACE_ETHN outside the main pathway. GSEM was conducted across income groups. Type I error was set at 0.05.

## Results

### Descriptive findings

Overall, a final sample of *N* = 4592 (Table 1) represented a population of 35,551,772 individuals aged ≥60 y, with 86% estimated as NHW, ~8% as NHB, ~2% as MA and ~4% as others (See Appendix IV). Notable differences in diet quality, cognition and health were observed in higher income groups compared with the lowest income group, defined by a cutoff 130% in the poverty income ratio.

**Table 1:** Baseline characteristics of selected participants by race/ethnicity among older adults (≥60 y), across income groups NHANES III, 1988–1994 (Unweighted *N* = 4,592; Weighted *N* = 35,551,773)^a^.

Selected participant characteristics	Lowest income group Poverty income ratio, PIR ≤ 130%	Middle income group 130%<PIR ≤ 300%	Highest income group PIR > 300%
**Unweighted sample**	***N*** = **1,451**	***N*** = **1,830**	***N*** = **1,311**
**Weighted population %**	**19.7%**	**40.9%**	**39.4%**
Sociodemographic characteristics			
Sex, % female	67.9 ± 1.8	57.8 ± 1.4	51.7 ± 1.1
Race/ethnicity			
Non-Hispanic White	69.7 ± 2.9	85.6 ± 1.4	94.2 ± 0.9
Non-Hispanic Black	19.0 ± 2.2	7.4 ± 0.8***	2.9 ± 0.5***
Mexican American	5.2 ± 0.6	1.9 ± 0.2***	0.8 ± 1.2***
Other race/ethnicity	6.7 ± 2.1	5.0 ± 1.0	2.1 ± 0.7**
Age (years)	72.3 ± 0.3	71.0 ± 0.4**	69.3 ± 0.4***
Urban/rural area of residence			
Urban	35.6 ± 5.0	36.0 ± 4.3	52.3 ± 6.0
Rural	64.4 ± 5.0	64.0 ± 4.3	47.7 ± 6.0**
Household size	2.01 ± 0.08	2.02 ± 0.04	2.01 ± 0.03
Marital status			
Never married	6.0 ± 1.5	4.3 ± 0.7	2.6 ± 0.6
Married	33.6 ± 2.4	58.5 ± 2.1*	74.1 ± 1.8***
Divorced	11.4 ± 1.8	5.5 ± 0.7	4.6 ± 0.7
Widowed	43.4 ± 2.1	29.0 ± 1.8	17.3 ± 1.4
Other	5.6 ± 1.0	2.7 ± 0.6	1.4 ± 0.5
Socioeconomic status			
Poverty income ratio	0.853 ± 0.020	2.100 ± 0.021***	4.871 ± 0.080***
Education, years	8.66 ± 0.21	10.63 ± 0.13***	12.96 ± 0.12***
**Socioeconomic status, SES z-score**	−0.546 ± 0.026	+0.027 ± 0.018***	1.064 ± 0.032***
Dietary quality			
1995-HEI total score	64.8 ± 0.6	67.5 ± 0.4**	71.1 ± 0.4***
MAR total score	67.1 ± 0.9	72.7 ± 0.5***	76.7 ± 0.5***
**Dietary quality, DIET z-score**	−0.15 ± 0.04	−0.11 ± 0.02***	+0.36 ± 0.02***
Nutritional biomarkers			
Folate, ng/mL	8.27 ± 0.36	9.27 ± 0.31*	10.11 ± 0.33***
Vitamin A, μg/dL	61.82 ± 1.01	64.16 ± 0.60*	65.17 ± 0.83**
Total carotenoids, μg/dL	80.51 ± 1.96	83.46 ± 1.65	90.06 ± 1.72**
Vitamin E, μg/dL	1342.3 ± 25.4	1405.7 ± 18.0*	1468.1 ± 24.2**
**Nutritional biomarkers, NUTR z-score**	−0.053 ± 0.031	+0.057 ± 0.023**	+0.163 ± 0.029***
Physical activity 0 = Less, 1 = Same, 2 = more			
Compare activity for past mo to past yr			
Less	27.6 ± 1.8	23.9 ± 1.5	22.5 ± 1.4
Same	66.5 ± 2.0	65.7 ± 1.7	66.4 ± 1.6**
More	5.9 ± 1.1	10.4 ± 1.0	11.0 ± 1.2**
Active compared with men/women your age			
Less	24.9 ± 2.1	17.2 ± 1.1	12.2 ± 1.2
Same	44.3 ± 2.0	41.9 ± 1.7*	38.9 ± 1.9***
More	30.8 ± 1.6	41.0 ± 1.6**	49.0 ± 2.0***
Active now compared with self 10 yrs ago			
Less	72.5 ± 2.0	66.5 ± 1.6	58.3 ± 1.8
Same	20.6 ± 1.8	26.7 ± 1.7*	34.1 ± 1.5***
More	6.9 ± 1.3	6.9 ± 1.0	7.6 ± 1.3
**Physical Activity, PA z-score**	−0.125 ± 0.031	−0.039 ± 0.018***	+0.159 ± 0.030***
Smoking			
# cigarettes/day	5.69 ± 0.43	6.26 ± 0.35	6.15 ± 0.41
Years smoked cigarettes	7.76 ± 0.61	8.23 ± 0.56	8.56 ± 0.72
**Smoking, SMOKING z-score**	+0.089 ± 0.042	+0.143 ± 0.037	0.145 ± 0.047
Alcohol consumption (g/d)	2.251 ± 0.437	4.329 ± 3.319***	7.466 ± 1.207***
**Alcohol consumption, ALCOHOL z-score**	−0.127 ± 0.032	+0.026 ± 0.037***	+0.256 ± 0.089***
Social support			
(1) In a typical week, how many times do you talk on the telephone with family, friends, or neighbors?	10.85 ± 0.54	10.36 ± 0.59	11.05 ± 0.40
(2) How often do you get together with friends or relatives; I mean things like going out together or visiting in each other’s homes? (per year)	127.7 ± 7.0	137.2 ± 9.7	113.6 ± 6.4
(3) About how often do you visit with any of your other neighbors, either in their homes or in your own? (per year)	113.00 ± 12.24	81.0 ± 6.7*	74.7 ± 5.7**
(4) How often do you attend church or religious services? (per year)	42.77 ± 3.11	45.34 ± 2.03	39.11 ± 2.69
(5) Altogether, how often do you attend meetings of the clubs or organizations (per year)	8.70 ± 1.42	13.40 ± 1.32*	20.76 ± 1.43***
**Social Support, SS z-score**	+0.023 ± 0.029	+0.018 ± 0.024	+0.009 ± 0.018
Health-related factors			
Self-rated health			
Excellent/Very Good	23.9 ± 2.0	33.1 ± 1.6	49.3 ± 2.3
Good	28.1 ± 2.4	35.8 ± 1.2	32.8 ± 1.9**
Fair/Poor	48.0 ± 2.2	31.1 ± 1.5***	17.9 ± 1.4***
Co-morbidity index	1.72 ± 0.05	1.66 ± 0.06	1.49 ± 0.05**
Allostatic load, AL score	2.92 ± 0.05	2.70 ± 0.08*	2.61 ± 0.05***
Body mass index, kg.m^−2^	27.2 ± 0.3	27.2 ± 0.2	26.7 ± 0.2
**Poor cardio-metabolic or general health; HEALTH z-score**	+0.109 ± 0.024	−0.025 ± 0.028**	−0.190 ± 0.022***
Cognitive performance test scores and index			
WR-CORR, (×−1)	−5.28 ± 0.04	−5.52 ± 0.02***	−5.69 ± 0.02***
WR-TRIALS	+0.083 ± 0.01	+0.039 ± 0.006**	+0.027 ± 0.005***
SR-CORR, (×−1)	−3.56 ± 0.08	−4.02 ± 0.04***	−4.47 ± 0.05***
MATH-INC	+1.42 ± 0.07	+0.62 ± 0.04***	+0.33 ± 0.03***
**COGN PCA score**	+0.272 ± 0.069	−0.308 ± 0.030***	−0.646 ± 0.026***
Cumulative incidence of all-cause dementia, %	39.8 ± 1.7	33.3 ± 1.7*	30.5 ± 1.4***
(*N* = 4,570)			

*AD* Alzheimer’s Disease, *ALCOHOL* alcohol consumption, z-score, *COGN* Cognitive performance principal component variable (4 measured variables), *DIET/NUTR* diet and nutritional biomarkers z-score variable (2 dietary quality measures and 4 nutritional biomarkers), *HEALTH* Health-related factors as mean of z-scores for allostatic load, self-rated health, co-morbidity index and body mass index, *LIFESTYLE* Lifestyle-related factors composed of social support, physical activity, diet/nutritional biomarkers, smoking and alcohol consumption using means of z-scores for related measured variables, *MA* Mexican American, *N* Number of participants, *N’* number of observations, *NHANES III* Third National Health and Nutrition and Examination Survey, *NHB* Non-Hispanic Blacks, *NHW* Non-Hispanic White, *PA* Physical activity z-score variable (3 measured variables), *RACE_ETHN* racial/ethnic contrast, *SES* Socioeconomic status mean of z-scores composed of poverty income ratio and education (years), *SMOKING* smoking z-score variable (2 measured variables), *SR-CORR* Story Recall test, correct items, *SS* Social Support z-score variable (5 measured variables), *WR-CORR* Word Recall Test, Correct Items, *WR-TRIALS* Word Recall Test, number of trials.

^a^Values are weighted means ± SEM or percent ± SEP, considering sampling design complexity (PSU and strata), across 5 imputations with 10 iterations.

^b^Design-based F-test accounting for design complexity in terms of sampling weights, PSU and stratum, using multinomial logit models for categorical variables and linear regression for continuous variables, taking NHW as the referent category.

*P < 0.05 **P < 0.01 ***P < 0.001 for null hypothesis of no difference between income groups, with lowest income group as the referent category, based on linear and multinomial logit models with income categories variable as the only categorical variable for continuous and categorical study characteristic, respectively, taking into account sampling design complexity.

### Cox models findings

Tables 2 and S1 shows Cox proportional hazards model findings with incident dementia as outcome, and race/ethnicity as primary exposure (NHW: referent group). In Table 2, NHB had overall a greater dementia risk compared with NHW (HR = 1.34, 95%CI: 1.15–1.55, *P* < 0.001, Model 1), a disparity explained by SES differences between NHB and NHW (Model 2, HR = 1.15, 95%CI: 1.01–1.34, *P* = 0.092). Conversely, within the lowest and middle-income groups, MA older adults had reduced dementia risk compared with their NHW counterparts, specifically in models adjusted for SES. The inverse association between SES and dementia risk (Model 2: per Standard Deviation, HR = 0.80, 95% CI:0.69–0.92, *P* = 0.002, Model 2)) was attenuated by adding lifestyle and health-related factors (Models 3 and 4), becoming non-significant when the “poor cognitive performance” principal components score was added (Model 5). Furthermore, lifestyle factors’ associations with dementia risk were contingent upon income group (social support *z*-score in the lowest income group, physical activity *z*-score in the middle-income group, and diet quality *z*-score in the highest income group, Model 4). In Model 5, diet quality’s inverse relationship with dementia risk within the upper-income group was attenuated upon “poor cognitive performance” *z*-score adjustment. Overall, and for all income groups combined, Non-White older adults did not differ in terms of dementia risk when compared with their NHW counterparts, according to Table S1. More generally, there was no heterogeneity detected across income groups in the race-dementia or SES-dementia associations (2-way interactions between race or SES and income groups, *p* > 0.05, Tables 2 and S1).

**Table 2:** Racial/ethnic disparities (NHB and MA vs. NHW) in incident all-cause dementia across income groups (≥60 y, Unweighted *N* = 4592; Weighted *N* = 35,551,773): Cox proportional hazards models; NHANES III, 1988–1994^a^.

	Overall		Lowest income group Poverty income ratio, PIR ≤ 130%		Middle income group 130%<PIR ≤ 300%		Highest income group PIR > 300%	
Unweighted sample	*N* = 4,570		*N* = 1,444		*N* = 1,817		*N* = 1,309	
	**Log**_**e**_**(HR)**	**(SE)**	**Log**_**e**_**(HR)**	**(SE)**	**Log**_**e**_**(HR)**	**(SE)**	**Log**_**e**_**(HR)**	**(SE)**
Model 1								
NHB vs. NHW	**+0.289**	(0.077)***	+0.035	(0.148)	+0.180	(0.186)	+0.022	(0.210)
MA vs. NHW	−0.031	(0.121)	−0.289	(0.162)	**−0.511**	(0.203)*	+0.091	(0.313)
OTHER vs. NHW	−0.203	(0.206)	−0.063	(0.260)	−0.655	(0.386)	−0.253	(0.476)
Model 2								
NHB vs. NHW	+0.139	(0.081)	−0.066	(0.169)	+0.104	(0.192)	+0.030	(0.222)
MA vs. NHW	−0.267	(0.140)	**−0.588**	(0.256)*	**−0.491**	(0.203)*	+0.092	(0.318)
OTHER vs. NHW	−0.370	(0.218)	−0.437	(0.336)	−0.588	(0.393)	−0.309	(0.457)
SES	**−0.224**	(0.069)**	−0.361	(0.203)	**−0.309**	(0.154)*	+0.054	(0.131)
Model 3								
NHB vs. NHW	+0.104	(0.080)	−0.018	(0.170)	+0.104	(0.192)	−0.055	(0.237)
MA vs. NHW	−0.264	(0.141)	**−0.597**	(0.256)*	**−0.491**	(0.203)*	+0.120	(0.298)
OTHER vs. NHW	−0.376	(0.229)	−0.460	(0.343)	−0.588	(0.393)	−0.307	(0.476)
SES	**−0.155**	(0.069)*	−0.311	(0.222)	−0.309	(0.154)	+0.089	(0.130)
*SMOKING*	*+0.097*	*(0.061)*	*+0.053*	*(0.111)*	*+0.099*	*(0.100)*	*+0.094*	*(0.102)*
* SS*	*−0.031*	*(0.077)*	***−0.418***	(0.160)*	*+0.029*	*(0.120)*	*+0.125*	*(0.125)*
* NUTR*	*−0.015*	*(0.061)*	*−0.013*	*(0.099)*	*−0.078*	*(0.080)*	*+0.066*	*(0.127)*
* DIET*	***−0.109***	(0.047)*	*+0.047*	*(0.095)*	*−0.141*	*(0.072)*	***−0.201***	(0.096)*
* PA*	***−0.243***	(0.056)***	*−0.232*	*(0.139)*	***−0.260***	(0.088)**	*−0.187*	*(0.102)*
*ALCOHOL*	*−0.023*	*(0.041)*	*+0.020*	*(0.069)*	*−0.035*	*(0.061)*	*−0.008*	*(0.053)*
Model 4								
NHB vs. NHW	+0.075	(0.080)	−0.085	(0.177)	+0.103	(0.190)	−0.078	(0.234)
MA vs. NHW	−0.269	(0.140)	**−0.620**	(0.257)*	**−0.489**	(0.202)*	+0.117	(0.294)
OTHER vs. NHW	−0.356	(0.228)	−0.442	(0.333)	−0.577	(0.393)	−0.309	(0.466)
SES	**−0.143**	(0.069)*	−0.306	(0.216)	−0.296	(0.159)	+0.093	(0.129)
*SMOKING*	*+0.095*	*(0.060)*	*+0.058*	*(0.110)*	*+0.098*	*(0.096)*	*+0.092*	*(0.129)*
*SS*	*−0.028*	*(0.076)*	***−0.407***	(0.154)*	*+0.031*	*(0.120)*	*+0.124*	*(0.126)*
*NUTR*	*−0.017*	*(0.061)*	*−0.019*	*(0.100)*	*−0.079*	*(0.080)*	*+0.069*	*(0.126)*
*DIET*	***−0.105***	(0.047)*	*+0.057*	*(0.093)*	*−0.138*	*(0.073)*	***−0.199***	(0.097)*
*PA*	***−0.216***	(0.058)**	*−0.203*	*(0.140)*	***−0.239***	(0.097)*	*−0.167*	*(0.104)*
*ALCOHOL*	*−0.020*	*(0.039)*	*+0.021*	*(0.069)*	*−0.032*	*(0.059)*	*−0.005*	*(0.051)*
HEALTH	+0.139	(0.073)	+0.183	(0.125)	+0.088	(0.128)	+0.102	(0.086)
Model 5								
NHB vs. NHW	−0.056	(0.082)	−0.157	(0.184)	−0.010	(0.184)	−0.131	(0.251)
MA vs. NHW	**−0.372**	(0.147)*	**−0.627**	(0.261)*	**−0.581**	(0.225)*	+0.026	(0.312)
OTHER vs. NHW	−0.364	(0.215)	−0.425	(0.311)	−0.582	(0.391)	−0.346	(0.465)
SES	−0.078	(0.066)	−0.200	(0.222)	−0.134	(0.159)	+0.124	(0.127)
*SMOKING*	*+0.097*	*(0.059)*	*+0.075*	*(0.107)*	*+0.087*	*(0.091)*	*+0.089*	*(0.099)*
*SS*	*−0.004*	*(0.080)*	***−0.380***	(0.156)*	*+0.048*	*(0.123)*	*+0.141*	*(0.136)*
*NUTR*	*−0.011*	*(0.061)*	*+0.015*	*(0.099)*	*−0.064*	*(0.080)*	*+0.062*	*(0.130)*
*DIET*	***−0.096***	(0.046)*	*+0.066*	*(0.093)*	*−0.134*	*(0.070)*	*−0.188*	*(0.100)*
*PA*	***−0.190***	(0.060)**	*−0.179*	*(0.137)*	***−0.223***	(0.097)*	*−0.141*	*(0.107)*
*ALCOHOL*	*−0.015*	*(0.038)*	*+0.018*	*(0.068)*	*−0.033*	*(0.058)*	*+0.006*	*(0.051)*
*HEALTH*	*+0.141*	*(0.072)*	*+0.184*	*(0.123)*	*+0.084*	*(0.128)*	*+0.113*	*(0.087)*
*COGN*	***+0.177***	(0.025)***	***+0.110***	(0.047)*	***+0.238***	(0.042)***	***+0.198***	(0.072)***

*ALCOHOL* alcohol consumption, z-score, *COGN* Cognitive performance principal component variable (4 measured variables), *DIET/NUTR* diet and nutritional biomarkers z-score variable (2 dietary quality measures and 4 nutritional biomarkers), *HEALTH* Health-related factors as mean of z-scores for allostatic load, self-rated health, co-morbidity index and body mass index, *HR* Hazard Ratio, *LIFESTYLE* Lifestyle-related factors composed of social support, physical activity, diet/nutritional biomarkers, smoking and alcohol consumption using means of z-scores for related measured variables, *MA* Mexican American, *N* Number of participants, *N’* number of observations, *NHANES III* Third National Health and Nutrition and Examination Survey, *NHB* Non-Hispanic Blacks, *NHW* Non-Hispanic White, *PA* Physical activity z-score variable (3 measured variables), *RACE_ETHN* racial/ethnic contrast, *SES* Socioeconomic status mean of z-scores composed of poverty income ratio and education (years), *SMOKING* smoking z-score variable (2 measured variables), *SS* Social Support z-score variable (5 measured variables). See Methods section for more details.

^a^Values are β ± SE (Log_e_(HR)), considering sampling design complexity (PSU and strata), across 5 imputations with 10 iterations.

Model 1: adjusted for age and sex; Model 2: adjusted for demographic factors other than age or sex, and for SES score; Model 3: Model 2 further adjusted for lifestyle-related factors (average of z-scores of measured variables for SMOKING, ALCOHOL, DIET, NUTR, SS and PA); Model 4: Model 3 + health-related factors (HEALTH score); Model 5: Full model with cognitive test PCA score.

^b^*P* < 0.05 for sex×RACE_ETHN interaction in unstratified model. ^c^ *P* < 0.05 for POVSTAT×SES interaction in unstratified model.

*P < 0.05 **P < 0.01 ***P < 0.001 for null hypothesis of Log_e_(HR) = 0.

### GSEM findings

**Fig. 1:**
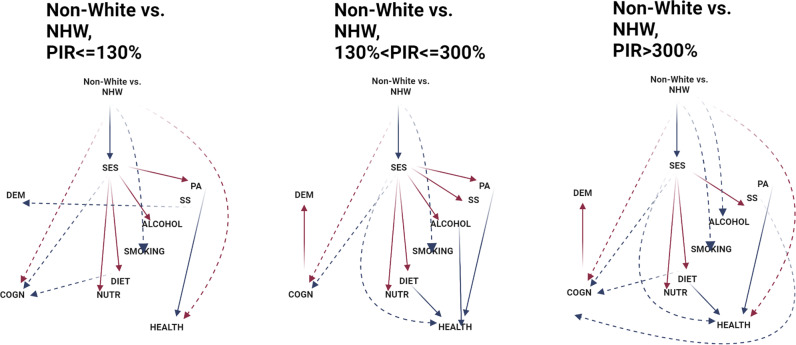
GSEM model findings for Non-White vs. NHW racial/ethnic contrast vs. DEMENTIA, NHANES III (1988–1994): Final eligible sample across income groups (*N* = 4592). ALCOHOL alcohol consumption, z-score, COGN Cognitive performance principal component variable (4 measured variables), DIET/NUTR diet and nutritional biomarkers z-score variable (2 dietary quality measures and 4 nutritional biomarkers), HEALTH Health-related factors as mean of z-scores for allostatic load, self-rated health, co-morbidity index and body mass index, LIFESTYLE Lifestyle-related factors composed of social support, physical activity, diet/nutritional biomarkers, smoking and alcohol consumption using means of z-scores for related measured variables, MA Mexican American, N Number of participants, N’ number of observations, NHANES III Third National Health and Nutrition and Examination Survey, NHB Non-Hispanic Blacks, NHW Non-Hispanic White, PA Physical activity z-score variable (3 measured variables), RACE_ETHN racial/ethnic contrast, SES Socioeconomic status mean of z-scores composed of poverty income ratio and education (years), SMOKING smoking z-score variable (2 measured variables), SS Social Support z-score variable (5 measured variables), TE Total effect; See Methods section for more details. Plain arrows are statistically significant associations (p < 0.05) within the hypothesized pathway; Dashed arrows are statistically significant associations (*p* < 0.05) outside the hypothesized pathway; Red arrows are for positive (+) associations; Blue arrows are for inverse (−) associations.

Table 3 and Fig. 1 show GSEM model findings for Non-White vs. NHW racial/ethnic contrast in relation to incident all-cause dementia, across income groups. In a reduced GSEM model (with exogenous covariates only), no effect of race/ethnicity on dementia was detected. In contrast, in another reduced model with exogenous variables that included SES as the only potential mediator, the total effect of SES was statistically significant in the middle-income group. Results from the full model shows that race/ethnicity contrast (Non-White vs. White) was associated with lower SES, which in turn was linked to poorer cognitive performance and higher dementia risk, with other mediators potentially at play that differ across income groups, in some groups influencing dementia risk directly rather than through cognitive performance. Most notably, in the highest income group, RACE_ETHN (Non-White vs. NHW)→SES(−)→[DIET( + ) or SS( + )]→COGN(−)→DEMENTIA( + ) were two main indirect pathways observed that included two lifestyle factors of diet and social support. These pathways suggested that Non-White adults had lower SES compared to White adults, a difference that determined poorer diet quality and/or less social support, which were directly associated with poorer cognitive performance predictive of dementia risk. Nevertheless, two dominant pathways in the two uppermost income groups were RACE_ETHN→SES(−)→COGN(−)→DEMENTIA( + ) and RACE_ETHN→COGN( + )→DEMENTIA( + ), while in the lowest income group there was no pathway linking race/ethnicity or SES to dementia, as indicated by the non-significant association between COGN and DEMENTIA in that group. Nevertheless, in the lowest income group, SS had a direct inverse association with dementia risk. It is worth noting that in all 3 income groups, HEALTH, reflective of poor cardio-metabolic and general health, had no direct relationship with cognitive performance or dementia risk.

**Table 3:** Pathways from race/ethnicity (Non-White vs. NHW) to incident all-cause dementia among older adults across income groups (Age_base_: 60 + y) through modifiable risk factors and cognitive performance; NHANES III, 1988–1994^a^.

	Lowest income group Poverty income ratio, PIR ≤ 130%		Middle income group 130%<PIR ≤ 300%		Highest income group PIR > 300%	
	β	(SE), *p*	β	(SE), *p*	β	(SE), *p*
*Main pathway*						
RACE_ETHN→SES (β_12_)	**−0.335**	**(0.045),** ***p*** < **0.001**	**−0.291**	**(0.045), p** < **0.001**	**−0.186**	**(0.056),** ***p*** = **0.002**
SES→SS (β_23_)	+0.078	(0.075), *p* = 0.31	**+0.197**	**(0.039),** ***p*** < **0.001**	**+0.085**	**(0.034),*p*** = **0.017**
SES→PA(β_24_)	**+0.171**	**(0.082),** ***p*** = **0.043**	**+0.256**	**(0.070),** ***p*** = **0.001**	+0.050	(0.044),*p* = 0.27
SES→DIET(β_25_)	**+0.414**	**(0.099),** ***p*** < **0.001**	**+0.236**	**(0.076),** ***p*** = **0.004**	**+0.193**	**(0.045),** ***p*** < **0.001**
SES → NUTR (β_26_)	**+0.143**	**(0.059),** ***p*** = **0.020**	**+0.183**	**(0.065),** ***p*** = **0.007**	**+0.118**	**(0.043),** ***p*** = **0.010**
SES → SMOKING (β_27_)	+0.002	(0.118), *p* = 0.99	−0.110	(0.116), *p* = 0.36	−0.108	(0.077),*p* = 0.18
SES → ALCOHOL (β_28_)	**+0.216**	**(0.082),** ***p*** = **0.011**	**+0.205**	**(0.086),** ***p*** = **0.021**	−0.090	(0.084)*,p* = 0.29
SS → HEALTH (β_39_)	+0.003	(0.048), *p* = 0.94	−0.037	(0.027), *p* = 0.18	+0.011	(0.042)*,p* = 0.81
PA → HEALTH (β_49_)	**−0.193**	**(0.050),** ***p*** < **0.001**	**−0.237**	**(0.029),** ***p*** < **0.001**	**−0.218**	**(0.034),*p*** < **0.001**
DIET → HEALTH (β_59_)	−0.039	(0.024), *p* = 0.11	−0.035	(0.027), *p* = 0.21	−0.034	(0.025),*p* = 0.18
NUTR → HEALTH (β_69_)	−0.038	(0.041), *p* = 0.36	+0.247	(0.029), *p* = 0.39	−0.022	(0.033), *p* = 0.51
SMOKING → HEALTH (β_79_)	−0.008	(0.036), *p* = 0.82	+0.010	(0.027), *p* = 0.71	−0.045	(0.028),*p* = 0.14
ALCOHOL → HEALTH (β_89_)	−0.025	(0.018), *p* = 0.17	**−0.041**	**(0.015),** ***p*** = **0.008**	−0.014	(0.019),*p* = 0.46
HEALTH → COGN (β_910_)	−0.024	(0.099), *p* = 0.81	−0.011	(0.051), *p* = 0.84	−0.001	(0.035),*p* = 0.98
COGN → DEMENTIA (β_1011_)	+0.073	(0.044), *p* = 0.11	**+0.203**	**(0.046),** ***p*** < **0.001**	**+0.180**	**(0.060),** ***p*** = **0.005**
*Selected direct effects on final outcomes*						
RACE_ETHN→DEMENTIA (β_111_)	−0.196	(0.167), *p* = 0.25	−0.216	(0.201), *p* = 0.29	−0.063	(0.200), *p* = 0.75
SES → DEMENTIA (β_211_)	−0.216	(0.186), *p* = 0.26	−0.157	(0.152), *p* = 0.31	+0.133	(0.138),*p* = 0.34
SS → DEMENTIA (β_311_)	**−0.444**	**(0.136),** ***p*** = **0.002**	+0.022	(0.113), *p* = 0.84	+0.171	(0.125),*p* = 0.18
PA → DEMENTIA (β_411_)	−0.178	(0.130), *p* = 0.18	−0.139	(0.095), *p* = 0.15	−0.143	(0.102),*p* = 0.17
DIET → DEMENTIA (β_511_)	+0.099	(0.094), *p* = 0.30	−0.119	(0.076), *p* = 0.12	−0.097	(0.104),*p* = 0.35
NUTR → DEMENTIA (β_611_)	−0.010	(0.094), *p* = 0.92	−0.054	(0.089), *p* = 0.51	+0.044	(0.121),*p* = 0.72
SMOKING → DEMENTIA (β_711_)	−0.051	(0.113), *p* = 0.66	+0.032	(0.101), *p* = 0.75	−0.010	(0.101),*p* = 0.92
ALCOHOL→ DEMENTIA (β_811_)	−0.059	(0.074), *p* = 0.43	−0.008	(0.055), *p* = 0.88	+0.015	(0.046)*,p* = 0.75
HEALTH → DEMENTIA (β_911_)	−0.004	(0.122), *p* = 0.97	−0.017	(0.130), *p* = 0.89	−0.064	(0.091),*p* = 0.49
*Other effects between endogenous variables*						
SES→HEALTH (β_29_)	−0.036	(0.084), *p* = 0.67	**−0.153**	**(0.057),** ***p*** = **0.010**	**−0.098**	**(0.044),*p*** = **0.030**
SES→COGN (β_210_)	**−0.995**	**(0.126),** ***p*** < **0.001**	**−0.570**	**(0.073),** ***p*** < **0.001**	**−0.184**	**(0.038),*p*** < **0.001**
SS→COGN (β_310_)	−0.096	(0.090), *p* = 0.29	−0.020	(0.047), *p* = 0.67	**−0.113**	**(0.046),*p*** = **0.019**
PA→COGN (β_410_)	−0.122	(0.086), *p* = 0.16	−0.079	(0.048), *p* = 0.11	−0.016	(0.041)*,p* = 0.69
DIET→COGN (β_510_)	**−0.125**	**(0.053),** ***p*** = **0.023**	−0.026	(0.045)*, p* = 0.57	**−0.073**	**(0.034),*p*** = **0.036**
NUTR→COGN (β_610_)	−0.022	(0.068), *p* = 0.74	−0.084	(0.044), *p* = 0.059	−0.019	(0.040),*p* = 0.64
SMOKING→COGN (β_710_)	−0.117	(0.059), *p* = 0.057	+0.016	(0.047), *p* = 0.74	+0.021	(0.025), *p* = 0.40
ALCOHOL→COGN (β_810_)	−0.003	(0.041), *p* *=* 0.95	−0.016	(0.024), *p* = −0.50	−0.039	(0.021),*p* = 0.072
*Other direct effects of race*						
RACE_ETHN→SS (β_13_)	+0.061	(0.074), *p* = 0.41	+0.122	(0.073), *p* = 0.11	+0.100	(0.077),*p* = 0.20
RACE_ETHN→PA(β_14_)	−0.098	(0.085), *p* = 0.26	+0.111	(0.090), *p* = 0.23	+0.063	(0.106),*p* = 0.56
RACE_ETHN→DIET(β_15_)	−0.060	(0.102), *p* = 0.56	−0.132	(0.067), p = 0.054	−0.168	(0.104),*p* = 0.12
RACE_ETHN→NUTR(β_16_)	−0.053	(0.062), *p* = 0.40	−0.055	(0.055), *p* = 0.32	+0.094	(0.136),*p* = 0.49
RACE_ETHN→SMOKING(β_17_)	**−0.223**	**(0.100),** ***p*** = **0.036**	**−0.305**	**(0.111),** ***p*** = **0.019**	**−0.300**	**(0.126),*p*** = **0.028**
RACE_ETHN→ALCOHOL(β_18_)	+0.133	(0.081), *p* = 0.11	−0.140	(0.131), *p* = 0.29	**−0.345**	**(0.140),*p*** = **0.017**
RACE_ETHN→HEALTH(β_19_)	**+0.140**	**(0.059),** ***p*** = **0.024**	−0.007	(0.054), *p* = 0.90	**+0.174**	**(0.052),*p*** = **0.003**
RACE_ETHN→COGN(β_110_)	**+0.355**	**(0.126),** ***p*** = **0.007**	**+0.280**	**(0.103),** ***p*** = **0.010**	**+0.321**	**(0.088),*p*** = **0.001**
TOTAL EFFECT OF RACE_ETHN	−0.043	(0.161), *p* = 0.79	−0.037	(0.190), *p* = 0.85	−0.011	(0.190), *p* = 0.95
*INDIRECT EFFECTS OF RACE_THN*						
RACE_ETHN → SES → DEMENTIA (β_A_)	+0.065	(0.055)	+0.047	(0.042)	−0.026	(0.028)
RACE_ETHN → SES → LIFESTYLE → DEMENTIA (β_B_)	+0.007	(0.022)	+0.022	(0.012)	+0.0014	(0.0051)
RACE_ETHN → SES → LIFESTYLE → HEALTH → DEMENTIA (β_C_)	+0.0000	(0.0020)	−0.0006	(0.0030)	−0.0003	(0.0005)
RACE_ETHN → SES → LIFESTYLE → HEALTH → COGN → DEMENTIA (β_D_)	−0.0000	(0.0001)	−0.0001	(0.0003)	+0.0000	(0.00002)
RACE_ETHN → SES → LIFESTYLE→ COGN → DEMENTIA (β_E_)	+0.0020	(0.0011)	**+0.0030**	(0.0013)*	+0.0009	(0.0005)
RACE_ETHN → SES → COGN → DEMENTIA (β_F_)	+0.0240	(0.0148)	**+0.0340**	(0.0088)***	**+0.0061**	0.0027*
TOTAL EFFECT OF SES	−0.303	(0.158), *p* = 0.064	**−0.360**	**(0.154),** ***p*** = 0.025*	+0.096	(0.130), *p* = 0.47
*INDIRECT EFFECTS OF SES*						
SES → LIFESTYLE → DEMENTIA (β_G_)	−0.0195	(0.0650)	−0.0751	(0.0436)	−0.0072	(0.0269)
SES → LIFESTYLE → HEALTH → DEMENTIA (β_H_)	+0.0000	(0.0054)	+0.0021	(0.011)	+0.0013	(0.0024)
SES → LIFESTYLE → HEALTH → COGN→DEMENTIA (β_I_)	+0.0001	(0.0030)	+0.0001	(0.0008)	+0.0000	(0.0001)
SES → LIFESTYLE → COGN→DEMENTIA (β_J_)	**−0.0061**	(0.0030)*	**−0.0100**	(0.0045)*	−0.0045	(0.0027)
SES → COGN→DEMENTIA (β_K_)	−0.073	(0.213)	**−0.117**	(0.025)***	**−0.033**	(0.013)***

*AD* Alzheimer’s Disease, *ALCOHOL* alcohol consumption, z-score, C*O*GN Cognitive performance principal component variable (4 measured variables), *DIET/NUTR* diet and nutritional biomarkers z-score variable (2 dietary quality measures and 4 nutritional biomarkers), *HEALTH* Health-related factors as mean of z-scores for allostatic load, self-rated health, co-morbidity index and body mass index, *LIFESTYLE* Lifestyle-related factors composed of social support, physical activity, diet/nutritional biomarkers, smoking and alcohol consumption using means of z-scores for related measured variables, *MA* Mexican American, *N* Number of participants, *N’* number of observations, *NHANES III* Third National Health and Nutrition and Examination Survey, *NHB* Non-Hispanic Blacks, *NHW* Non-Hispanic White, *PA* Physical activity z-score variable (3 measured variables), *RACE_ETHN* racial/ethnic contrast, *SES* Socioeconomic status mean of z-scores composed of poverty income ratio and education (years), *SMOKING* smoking z-score variable (2 measured variables), *SS* Social Support z-score variable (5 measured variables). See Methods section for more details.

^a^Values are path coefficients β ± SE or nonlinear combinations of path coefficients to compute selected indirect effects, considering sampling design complexity (PSU and strata), across 5 imputations with 10 iterations. For indirect effects, Rubin’s rule was applied in order to pool estimates across the 5 imputations.

*P < 0.05 **P < 0.01 ***P < 0.001 for null hypothesis of β = 0.

Table 4 shows total and indirect pathways between SES and dementia risk overall and across income groups in the GSEM models. Generally, 3 pathways were found explaining >55% of the total effect of SES on dementia risk (total effect (TE) = −0.160 ± 0.067, *p* = 0.022), namely SES→LIFESTYLE→DEMENTIA (indirect effect (IE) = −0.041 ± 0.014, *p* = 0.004), SES→LIFESTYLE→COGN→DEMENTIA( + ) (IE = −0.006 ± 0.001, *p* < 0.001), SES→COGN→DEMENTIA( + ) (IE = −0.040 ± 0.008, *p* < 0.001), indicating a net inverse indirect relationship between socio-economic status and dementia being explained by cognitive performance being greater with higher SES, lifestyle factors directly impacting dementia risk after being determined by SES, or a combination of cognitive performance and lifestyle factors being impacted by SES in a serial fashion, whereby lifestyle factors are antecedent to cognition. Importantly, the last two remained statistically significant or marginally significant in the uppermost income groups.

**Table 4:** Total, direct and selected indirect effects of modifiable risk factors and cognitive performance from SES to incident all-cause dementia among older adults, overall (Age_base_: 60 + y); NHANES III, 1988–1994^a^.

	β	(SE), *p*
**TOTAL EFFECT OF SES**	**−0.160**	**(0.067),** ***p*** = 0.022*
**DIRECT EFFECT OF SES**	−0.050	(0.070), *p* = 0.41
*SELECTED INDIRECT EFFECTS OF SES*		
SES → LIFESTYLE → DEMENTIA (β_G_)	**−0.0397**	(0.0144)**
SES → LIFESTYLE → HEALTH → DEMENTIA (β_H_)	+0.0004	(0.0032)
SES → LIFESTYLE → HEALTH → COGN→DEMENTIA (β_I_)	+0.0001	(0.0002)
SES → LIFESTYLE → COGN→DEMENTIA (β_J_)	**−0.0067**	(0.0012)***
SES → COGN→DEMENTIA (β_K_)	**−0.0493**	(0.0087)***

*AD* Alzheimer’s Disease, *ALCOHOL* alcohol consumption, z-score, *COGN* Cognitive performance principal component variable (4 measured variables), *DIET*/*NUTR* diet and nutritional biomarkers z-score variable (2 dietary quality measures and 4 nutritional biomarkers), *HEALTH* Health-related factors as mean of z-scores for allostatic load, self-rated health, co-morbidity index and body mass index, *LIFESTYLE* Lifestyle-related factors composed of social support, physical activity, diet/nutritional biomarkers, smoking and alcohol consumption using means of z-scores for related measured variables, *MA* Mexican American, *N* Number of participants, *N’* number of observations, *NHANES III* Third National Health and Nutrition and Examination Survey, *NHB* Non-Hispanic Blacks, *NHW* Non-Hispanic White, *PA* Physical activity z-score variable (3 measured variables), *RACE_ETHN* racial/ethnic contrast; *SES* Socioeconomic status mean of z-scores composed of poverty income ratio and education (years), *SMOKING* smoking z-score variable (2 measured variables); SS Social Support z-score variable (5 measured variables). See Methods section for more details.

^a^Values are path coefficients β ± SE or nonlinear combinations of path coefficients to compute selected indirect effects, considering sampling design complexity (PSU and strata), across 5 imputations with 10 iterations. For indirect effects, Rubin’s rule was applied in order to pool estimate across the 5 imputations.

*P < 0.05 **P < 0.01 ***P < 0.001 for null hypothesis of β = 0.

## Discussion

### Summary of findings

This study tested racial/ethnic and socioeconomic differences in dementia incidence, overall and across income groups, using a nationally representative sample of older adults with Medicare linkage and mortality validation through NDI. Non-Hispanic Black adults had higher risk of dementia compared to their NHW counterparts, in age and sex-adjusted Cox regression models (HR = 1.34, 95%CI: 1.15–1.55, *P* < 0.001), an association attenuated in the SES-adjusted model (HR = 1.15, 95%CI: 1.01–1.34, *P* = 0.092). SES was inversely related to dementia risk (per SD, HR = 0.80, 95% CI:0.69–0.92, *P* = 0.002, Model 2), mainly within the middle-income group. Within the lowest and middle-income groups and SES-adjusted models, Mexican Americans were at lower all-cause dementia risk compared with NHW. GSEM models detected 3 pathways explaining >55% total socio-economic status effect on dementia risk (Total effect = −0.160 ± 0.067, *p* = 0.022), namely socio-economic status impacting lifestyle, which in turn has an effect on dementia risk (SES→LIFESTYLE→DEMENTIA); Indirect effect (IE) = −0.041 ± 0.014, *p* = 0.004), another pathway going also through cognitive performance (SES→LIFESTYLE→COGN→DEMENTIA, IE = −0.006 ± 0.001, *p* < 0.001), and a third pathway going directly from socioeconomic status into cognition which then is related to dementia risk (SES→COGN→DEMENTIA;IE = −0.040 ± 0.008, *p* < 0.001), with the last two remaining significant or marginally significant in the uppermost income groups. Diet and social support were among key lifestyle factors involved in socio-economic disparities in dementia incidence.

### Previous studies

Previous studies point to major racial disparities in AD and related dementias [5–8]. For instance, a recent study showed that in a multi-ethnic cohort, the age-standardized diagnostic incidence rate of all-cause dementia was higher among African Americans (22.9 in women, 21.5 in men) and Native Hawaiians (19.3, 19.4) compared to White adults (16.4, 15.5), while being comparable among Latinos (16.8, 14.7) and significantly lower among Japanese Americans (14.8, 13.8), and Filipinos (12.5, 9.7) [6]. In contrast, an earlier study focused on AD patients and using data from over thirty U.S. Alzheimer’s Disease Centers (1984–2005), indicated that African American and Latino Alzheimer disease (AD) patients may have longer survival when compared with their White counterparts, with neuropathology not explaining this difference in survival [7]. Our recent study shows that, in sex-stratified analyses, incident all-cause dementia among older adults in the US was significantly greater among NHB women compared to NHW women, while Mexican-American women had a reduced AD risk compared to their NHW counterpart, particularly upon adjustment for SES and other upstream factors [8]. This study indicated that SES mediated a large portion of the NHB-NHW women disparity in dementia, in combination with several other lifestyle factors, particularly diet and physical activity [8]. Other studies have suggested that a socio-economic gradient in dementia incidence may play a major role in racial/ethnic disparities in this health outcome [9–15]. Examining several related outcomes, one of these studies conducted among 859 older Catholic clergy members without dementia at baseline, indicated that early life socioeconomic level was related to cognitive performance in late life without being associated with the rate of cognitive decline or incidence of AD [14]. Another study shows that early life educational attainment’s association with AD was not mediated by later life socio-economic mobility, highlighting the importance of cognitive reserve in the etiology of AD [11]. More recently, the beneficial effect of education has been ascribed to reduced cognitive adverse effects of tau accumulation as imaged with in vivo positron emission tomography, one of two hallmarks of AD pathology, with higher education [15]. Our present study indicated that a measure combining early-life cognitive reserve and later-life socioeconomic mobility was associated with reduced all-cause dementia risk through later-life cognition particularly in the uppermost income groups.

Moreover, previous studies support our findings, specifically showing the increases in risky behaviors, poorer diet quality and lack of access to quality resources with lower socioeconomic status [3, 4, 16]. Lack of access to resources is a structural determinant that links low socio-economic status with dementia especially among historically marginalized groups [18]. Both low socio-economic status and lack of social support have also been linked to additive chronic stress. Accumulation of allostatic load is a mechanism by which chronic stress, such as low socio-economic status is thought to cause cognitive dysfunction [24]. Considering social support is associated with lower allostatic load, lack of social support is expected to potentially trigger cognitive dysfunction [18, 21, 22]. Additionally, with low socioeconomic status, chronic stress may lead to potential maladaptive responses, resulting in neuroendocrine, autonomic, and behavioral modifications. These modifications are thought to be associated with poor cognitive function. Researchers have found the prefrontal cortex to be negatively affected by chronic stress, a byproduct of low SES [27]. These cortical changes can also be attributed to cognitive dysfunction. Thus, low socioeconomic status is linked to a complex interplay of biological, physiological, and environmental factors which, in turn, results in cognitive dysfunction.

In our study, the connection between race and dementia was mediated by diet quality and social support especially in the uppermost income group. Dietary measures and social support are both important components of social determinants of health. According to one study, greater childhood social support predicted higher educational attainment and better physical and emotional health in adulthood, which were each associated with better memory [3]. Using data from African Americans who participated in the Brain and Health sub-study of the Baltimore Experience Corps Trial, previous researchers found higher enriching early-life activity score including a supportive environment was linked to favorable outcomes in cognitive function [48]. Aside from mediation by social support, evidence for mediation by dietary quality is demonstrated through previous studies [48, 49]. For instance, there was a racial gap between dietary quality in urban dwelling Caucasian versus African American adults within the HANDLS cohort [49]. Evidence leads credence to the theory that dietary quality compounded with disparities in race result in increased cognitive dysfunction.

We found that the relationship between race and dementia was mediated by SES. Racial residential segregation is compounded with economic residential segregation as reflected by geographic socioeconomic variables like Area Deprivation Indices (ADI) when understanding health outcomes like dementia [49]. Among older Caribbean-born African American individuals compared to US-born African American individuals, analyses showed cognitive variation according to SES modifying race [50]. However, differences in neuropsychological test performance between the two groups was explained by higher quality of education among the Caribbean-born African American cohort. Moreover, according to the Canadian Community Health Survey, from a study sample of 20,646 people ≥60 years, SES mediated racial gap in cognitive functioning [51]. This national study demonstrates the importance of understanding that social determinants such as race and SES have a combined negative effect on cognitive function.

Another main finding was that there was a direct effect of race/ethnicity on cognitive performance not explained by SES in the two highest income groups. While previous studies did not stratify by income groups, they reported a similar finding over a wide range of income categories. Some brain autopsy studies reported more senile plaques and neurofibrillary tangles that are characteristics of AD in African American than among Caucasian individuals with dementia before death [39], and higher AD frequency in Caucasian than African American individuals [52]. The direct impact of race on dementia risk may be the consequence of other environmental and psychosocial factors that were not accounted for in this study.

Another finding was that cognitive performance measured at the baseline MEC visit is not always predictive of future dementia risk, especially in the lowest income group whereby cognitive performance had no association with dementia. Using the Whitehall II cohort study, researchers found no significant association between cognitive impairment and dementia, especially in early old age [53]. Cognitive impairment was defined using both cognitive performance and decline. Applying a comprehensive case definition is important in conducting research and understanding how demographic variables impact cognitive performance.

### Strengths and limitations

Our study has several strengths. First, the large sample size sufficiently powered our analyses to detect mediation effects across strata defined by the intersection of demographic characteristics. We used a nationally representative sample with Medicare linkage which allowed us to combine detailed information about respondents with their medical diagnoses. Studies strictly using claims data rely on accurate demographic reporting during patient encounters [54] and generally exclude microlevel, nonmedical information. Classifying respondents with cognitive impairment using cognitive tasks is prone to measurement bias due to varying thresholds among demographic subgroups with different educational attainment and literacy. In addition, clinical diagnosis of dementia relies on subjective assessment methods in addition to objective measurement. Thus, by combining a large, population-based survey with Medicare linkage, we overcame limitations typically experienced in prior work that relies on population-based survey or medical claims record data. Furthermore, the depth of NHANES allowed us to test various pathways spanning multiple domains of risk.

Limitations of our study include those typically reported in observational studies, including residual confounding, measurement error, and potential selection bias due to missing data on cognitive performance. However, the breadth of available covariates helps mitigate residual confounding concerns. Nevertheless, given restrictive methods as applied to imputed data, our study used average z-scores instead of principal components analysis predicted scores for most constructs, except for poor cognitive performance, a construct that determined the final sample size of the imputed dataset. In addition to the drawback of assuming equal weighting for measured variables for each construct, the variability was limited when only one or two measured variables were included to reflect that construct (e.g. ALCOHOL, DIET, SMOKING).

## Conclusions

This work builds on existing literature reporting racial/ethnic and socioeconomic disparities in dementia risk by identifying mediating factors between race/ethnicity and time to incident dementia, overall and within each income group. Although disease-modifying strategies and cures are lacking, compressing all-cause dementia risk closer towards the end of life can have marked individual- and population-level benefits [55]. We provide evidence for modifiable risk factors that may delay dementia onset and explain the SES-dementia relationship overall and across income groups. Our findings underscore the importance of lifestyle factors such as diet and social support for future observational and intervention studies.

## Supplementary information

ONLINE SUPPLEMENTARY MATERIALS

Figure S1

Figure S2
