# Supplementary material for: Pathways explaining racial/ethnic and socio-economic disparities in incident all-cause dementia among older US adults across income groups
Source: Transl Psychiatry. 2022 Nov 15;12:478. doi: 10.1038/s41398-022-02243-y (PMC9666623; doi:10.1038/s41398-022-02243-y)
Supplement: Supplementary file 1 — ONLINE SUPPLEMENTARY MATERIALS [file 41398_2022_2243_MOESM1_ESM.docx]

**ONLINE SUPPORTING MATERIALS**

**Pathways explaining racial/ethnic and socio-economic disparities in incident all-cause dementia among older US adults across income groups**

May A. Beydoun et. al.

**APPENDIX I: MEDICARE AND NDI LINAKAGE**

Administered by the Centers for Medicare and Medicaid (CMS), Medicare is the primary health insurance program for people ≥65 y, people <65y with specific disabilities, and all individuals with End-Stage Renal Disease (ESRD).^1^ Medicare enrollment and fee-for-service (FFS) claims data were linked to participants in the NHANES III by matching on Social Security Number (SSN), date of birth (month, day, year), and sex. Since 2006, CMS performed linkage on NHANES participants matching them with corresponding Medicare FFS annual claims data, and with Medicare Part D prescriptions.^1^ Annual files are available for Part A (inpatient, outpatient, Skilled Nursing Facility [SNF], hospice, or Home Health Agency [HHA]) and Part B (Carrier, Durable Medical Equipment).^1^ These files were analyzed to identify the first report of dementia and AD diagnoses for NHANES participants. Methods published elsewhere were used to estimate the earliest occurrence of the two incident outcomes of interest between 1991 and 1998.^1^ Restricted mortality data linked to NHANES III through the National Death Index was used to evaluate AD mortality risk through 2013 in all analyses. ^2^ Whereas AD was diagnosed using ICD-9 code 331.0 (any DX on the claim) from inpatient, Skilled Nursing Facilities (SNF), Home Health Agencies (HHA), and Health Options Program (HOP) or Carrier claims during a 3-year period, all-cause dementia was defined with one or more of the following diagnostic codes : 331.0, 331.1, 331.11, 331.19, 331.2, 331.7, 290.0, 290.10, 290.11, 290.12, 290.13, 290.20, 290.21, 290.3, 290.40, 290.41, 290.42, 290.43, 294.0, 294.1, 294.10, 294.11, 294.8 and 797.

**APPENDIX II: SES, LIFESTYLE AND HEALTH MEDIATORS**

*Socio-demographic factors and socio-economic status (SES)*

Key socio-demographic covariates included age (y), sex, race (Non-Hispanic white (NHW), Non-Hispanic black (NHB), Mexican-American (MA), other ethnicity (OTHER)); marital status (Never married, Married, Divorced, Widowed, Other), household size and urban-rural residence (Urban, Rural). Among SES factors, educational level (years completed); poverty income ratio (PIR) were selected.

*Lifestyle and social support factors*

Lifestyle factors include “substance abuse” operationalized as “alcohol consumption (g/d)” and drug use (Ever, Never); “nutritional factors” as 1995-Healthy Eating Index (1995-HEI) ranging from 0-100,^3^ and mean adequacy ratio score (MAR), ^4-6^ (**Tables II.1** and **II.2**); “Physical activity” measured with 3 items: (1) “Compare activity for past month to past year (less, same, more), (2): “Active compared with men/women your age” (less, same, more), and (3): “Active now compared with self, 10 years ago” (less, same, more); “Smoking” with two items: (1): “number of cigarettes smoked per day” (0 among non-smokers); (2): “years smoked cigarettes” (0 among non-smokers), drug and alcohol use. Five items were used for social support: “In a typical week, how many times do you” … (1) talk on the telephone with family, friends, or neighbors?”, (2) “get together with friends or relatives? (# per year)”, (3) “visit with any of your other neighbors, either in their homes or in your own? (# per year)”, (4) “attend church or religious services? (# per year)”, (5) “attend meetings of the clubs or organizations (# per year)”.

*Nutritional biomarkers and health-related factors*

Several nutritional biomarkers were included among potential confounders, namely serum 25-hydroxyvitamin D [25(OH)] and folate.

The “Health” construct was operationalized with self-rated health, co-morbidity index and allostatic load (AL) score. Self-rated health was defined as “Excellent” (referent), “Very good”, “Good”, “Fair” or “Poor.” The co-morbidity index is an unweighted sum of 14 binary self-reported conditions, including “arthritis“, “congestive heart failure“, “stroke“, “asthma“, “chronic bronchitis“, “emphysema“, “hay fever“, “cataracts“, “goiter“, “thyroid disease“, “lupus“, “gout“, “skin cancer”, and “other cancer”. The allostatic load (AL) total score (0-9) consists of 9 items which are described in details elsewhere.^7^ AL total score sums up cardiovascular (systolic and diastolic blood pressure, pulse rate), metabolic (total cholesterol, HDL-cholesterol, glycosylated Hb, sex-specific waist-to-hip ratio) and inflammatory (albumin and C-reactive protein (CRP)) risk indicators. Clinical criteria are summarized in **Table II.3**. Weight status was measured using body mass index (weight/squared-height, kg.m^-2^) categorized as: <18.5 (underweight), 18.5-24.9 (normal weight), 25-29.9 (overweight), ≥30 as obese.

Total cholesterol (mg/dl), HDL-cholesterol (mg/dl), CRP (mg/dl), albumin (g/dl) and glycosylated hemoglobin (%) were measured by laboratories using reference analytical methods (See Laboratory Procedures for NHANES III).^8^ Using standard protocols, waist-to-hip ratio, radial pulse (beats/min), and systolic and diastolic blood pressure (mmHg) were measured by trained examiners. Blood pressure was determined using a mercury sphygmomanometer.^8^ The arithmetic mean of three systolic and diastolic pressures was used in the analysis.

**Table II. 1. 1995-HEI**

|  | Criteria^1^ | |  |
| --- | --- | --- | --- |
| Components | <50y | ≥50y | Score |
| Grains | 9.1 servings/d | 7.4 servings/d | 10; 1 point less for each 10% less than intake required for full score |
|  |  |  | Range: 0-10 |
|  |  |  |  |
| Vegetables | 4.2 servings/d | 3.5 servings/d | Same as above |
|  |  |  |  |
| Fruit | 3.2 servings/d | 2.5 servings/d | Same as above |
|  |  |  |  |
| Milk | 2.0 servings/d | 2.0 servings/d | Same as above |
|  |  |  |  |
| Meat | 2.4 servings/d | 2.2 servings/d | Same as above |
|  |  |  |  |
| Total fat | ≤30% energy | ≤30% energy | 10 |
|  | 31-44% of energy | 31-44% of energy | 5 |
|  | ≥45% of energy | ≥45% of energy | 0 |
|  |  |  |  |
| Saturated fat | ≤10% of energy | ≤10% of energy | 10 |
|  | 11-14% of energy | 11-14% of energy | 5 |
|  | ≥15% of energy | ≥15% of energy | 0 |
|  |  |  |  |
| Cholesterol | <300 mg | <300 mg | 10 |
|  | 301-449 mg | 301-449 mg | 5 |
|  | ≥450 mg | ≥450 mg | 0 |
| Sodium | ≤2,400 mg | ≤2,400 mg | 10; 1 point less for each 10% less intake required for full score |
|  |  |  |  |
| Variety | Top 10% intake of sum of unique foods | Top 10% intake of sum of unique foods | Same as above |

*Abbreviations*: 1995-HEI=1995 Healthy Eating Index.

^1^ Based on 2,200 kcal for the <50 y category and 1,900 kcal for the ≥51 y category.

*Source*: ^3, 9^

**MAR scores**

RDAs of 16 vitamins and minerals were used to determine the nutrient adequacy ratio (NAR), using the following formula: NAR = [Subject’s daily intake of nutrient] / [RDA of nutrient]. An adjustment of an additional 35 mg Vitamin C must be applied to the RDA for participants who were current smokers.

The NAR of each nutrient is converted to a percentage, and percentages greater than 100 are truncated to 100. The total quality of the diet is then calculated from the NARs to form a mean adequacy ratio (MAR) using the following formula: MAR = [Sum of all 16 nutrient NARs]/16. The NAR and MAR for each day were calculated and averaged over the two days.

**Table II. 2.** Recommended Dietary Allowance (RDA) Values for Nutrients accessed from USDA DRI Tables to MAR Score

| **Nutrient** | **Men**  **19-30yrs** | **Men**  **31-50yrs** | **Men**  **51-70 yrs** | **Women**  **19-30yrs** | **Women**  **31-50yrs** | **Women**   - 1. **yrs** |
| --- | --- | --- | --- | --- | --- | --- |
| Vitamin A | 900 ug/day | 900 ug/day | 900 ug/day | 700 ug/day | 700 ug/day | 700 ug/day |
| Vitamin C | 90 mg/day | 90 mg/day | 90 mg/day | 75 mg/day | 75 mg/day | 75 mg/day |
| **Vitamin C for smokers** | 125 mg/day | 125 mg/day | 125 mg/day | 110 mg/day | 110 mg/day | 110 mg/day |
| Vitamin D | 15 ug/day | 15 ug/day | 15 ug/day | 15 ug/day | 15 ug/day | 15 ug/day |
| Vitamin E | 15 mg/day | 15 mg/day | 15 mg/day | 15 mg/day | 15 mg/day | 15 mg/day |
| Vitamin B6 | 1.3 mg/day | 1.3 mg/day | 1.7 mg/day | 1.3 mg/day | 1.3 mg/day | 1.5 mg/day |
| Vitamin B12 | 2.4 ug/day | 2.4 ug/day | 2.4 ug/day | 2.4 ug/day | 2.4 ug/day | 2.4 ug/day |
| Thiamin | 1.2 mg/day | 1.2 mg/day | 1.2 mg/day | 1.1 mg/day | 1.1 mg/day | 1.1 mg/day |
| Riboflavin | 1.3 mg/ day | 1.3 mg/ day | 1.3 mg/day | 1.1 mg/day | 1.1 mg/day | 1.1 mg/day |
| Niacin | 16 mg day | 16 mg day | 16 mg/day | 14 mg/day | 14 mg/day | 14 mg/day |
| Folate | 400 ug/day | 400 ug/day | 400 ug/day | 400 ug/day | 400 ug/day | 400 ug/day |
| Iron | 8 mg/day | 8 mg/day | 8 mg/day | 18 mg/day | 18 mg/day | 8 mg/day |
| Copper | 900 ug/day | 900 ug/day | 900 ug/day | 900 ug/day | 900 ug/day | 900 ug/day |
| Zinc | 11 mg/day | 11 mg/day | 11 mg/day | 8 mg/day | 8 mg/day | 8 mg/day |
| Calcium | 1,000 mg/day | 1,000 mg/day | 1,000 mg/day | 1,000 mg/day | 1,000 mg/day | 1,200 mg/day |
| Magnesium | 400 mg/day | 420 mg/day | 420 mg/day | 310 mg/day | 320 mg/day | 320 mg/day |
| Phosphorous | 700 mg/day | 700 mg/day | 700 mg/day | 700 mg/day | 700 mg/day | 700 mg/day |

*Abbreviations*: DRI=Dietary Recommended Intake; MAR=Mean Adequacy Ratio; NAR=Nutrient Adequacy Ratio; RDA=Recommended Dietary Allowance; USDA=United States Department of Agriculture.

ug= micrograms; mg= milligrams; g=grams

<http://www.nal.usda.gov/fnic/DRI/DRI_Tables/RDA_AI_vitamins_elements.pdf>

<http://ods.od.nih.gov/factsheets/VitaminC-HealthProfessional/>

**Allostatic Load (AL)**

A total AL score was calculated using a method described in a previous study.^7^ AL total score sums up cardiovascular (systolic and diastolic blood pressure, pulse rate), metabolic (total cholesterol, HDLcholesterol, glycosylated Hb, sex-specific waist-to-hip ratio) and inflammatory (albumin and C-reactive protein (CRP)) risk indicators. Clinical criteria summarized in Table I.2 were used to obtain risk indicators which were summed with equal weighting to compute total AL score (range: 0-9).

Total cholesterol (mg/dl), HDL-cholesterol (mg/dl), CRP (mg/dl), albumin (g/dl) and glycosylated hemoglobin (%) were measured by contract laboratories using reference analytical methods (See Laboratory Procedures for NHANES III).^8^ Using standard protocols, waist-to-hip ratio, radial pulse (beats/min), systolic and diastolic blood pressure (mmHg) were measured by trained examiners. Specifically, blood pressure measurements were taken using a mercury sphygmomanometer ^8^ The arithmetic mean of three systolic and diastolic pressures was used in this analysis.

**Table II.3 Allostatic load indicator criteria^7^**

|  | High-risk clinical |
| --- | --- |
| Albumin (g/dL) | < 3.8 ^10^ |
| C-reactive protein (mg/dL) | ≥ 0.3 ^11^ |
| Waist:Hip Ratio | >0.9 for men; > 0.85 for women ^12^ |
| Total cholesterol (mg/dL) | ≥240^13^ |
| HDL-C (mg/dL) | <40^13^ |
| Glycated hemoglobin (%) | ≥6.4^14, 15^ |
| Resting heart rate (beat/min) | ≥90^16^ |
| Systolic BP | ≥140^17^ |
| Diastolic BP | ≥90^17^ |

*Abbreviations*: BP=Blood Pressure; HDL=High Density Lipoprotein-Cholesterol

.

The measured variables used to compute each of the 8 constructs, namely SES, DIET, NUTR, SMOKING, ALCOHOL, SS, PA and HEALTH were regressed in their original form on their corresponding construct to obtain a predictive model that can be used for ease of interpretation. The results are shown in **Table II.4**.

**Table II.4 OLS regression analysis of the 8 constructs on their raw measured variables, NHANES III (1988-1994), final imputed sample (N=4,592)**

|  | β | (SE) | P |
| --- | --- | --- | --- |
| **MODEL 1** |  |  |  |
| Y=SES |  |  |  |
| X1=education (years) | 0.1139107 | 0.0000591 | <0.001 |
| X2=Poverty-income ratio | 0.2785572 | 0.0003625 | <0.001 |
| _Cons | -1.769242 | 0.0015884 | <0.001 |
| **MODEL 2** |  |  |  |
| Y=DIET |  |  |  |
| X1=HEI-1995 | +0.1353974 | (0.0000401) | 0.14 |
| X2=MAR | +0.0295001 | (0.0000247) | 0.006 |
| _Cons | -4.425294 | (0.002785) | 0.056 |
| **MODEL 3** |  |  |  |
| Y=NUTR |  |  |  |
| X1=Folate | +0.0348383 | (0.0000345) | <0.001 |
| X2=Vitamin A | +0.0138385 | (0.0000132) | 0.010 |
| X3=Total carotenoids | +0.0055232 | (0.000000) | 0.038 |
| X4=Vitamin E | +0.0004203 | (0.000000) | 0.009 |
| _Cons | -2.205515 | (0.0005885) | <0.001 |
| **MODEL 4** |  |  |  |
| Y=SMOKING |  |  |  |
| X1=number of cigarettes/day | +0.0561663 | (0.0001853) | <0.001 |
| X2=Years smoked | +0.0365893 | (0.0002457) | <0.001 |
| _Cons | -0.5125819 | (0.0036302) | <0.001 |
| **MODEL 5** |  |  |  |
| Y=ALCOHOL |  |  |  |
| X1=alcohol consumption | +0.0734859 | 0.0000513 | <0.001 |
| _Cons | -0.2926374 | 0.00008574 | <0.001 |
| **MODEL 6** |  |  |  |
| Y=SS |  |  |  |
| X1=SS1 | +0.0140336 | (0.0000000) | <0.001 |
| X2=SS2 | +0.0011773 | (0.0000000) | <0.001 |
| X3=SS3 | +0.0012602 | (0.0000000) | <0.001 |
| X4=SS4 | +0.0030534 | (0.0000000) | <0.001 |
| X5=SS5 | +0.0049120 | (0.0000000) | <0.001 |
| _Cons | -0.5955124 | 0.0002061 | <0.001 |
| **MODEL 7** |  |  |  |
| Y=PA |  |  |  |
| X1=PA1 | +0.6156738 | (0.0002388) | <0.001 |
| X2=PA2 | +0.4541654 | (0.0004136) | 0.063 |
| X3=PA3 | +0.543189 | (0.000211) | <0.001 |
| **MODEL 8** |  |  |  |
| Y=HEALTH |  |  |  |
| X1=SRH | +0.3078811 | (0.0000605) | <0.001 |
| X2=COMORBID | +0.1836388 | (0.0000648) | <0.001 |
| X3=AL | +0.1845449 | (0.0002233) | <0.001 |
| X4=BMI | +0.0485181 | (0.0000145) | <0.001 |
| _Cons | -2.758453 | (0.000833) | <0.001 |
| **MODEL 9** |  |  |  |
| Y=COGN PCA score |  |  |  |
| X1= WR-CORR, (×-1) | +0.576 | (0.00000) | <0.001 |
| X2=WR-TRIALS | +1.633 | (0.0000) | <0.001 |
| X3=SR-CORR, (×-1) | +0.309 | (0.00000) | <0.001 |
| X4=MATH-INC | +0.282 | (0.00000) | <0.001 |
| _cons |  |  |  |

*Abbreviations*: COGN=Poor Cognitive performance PCA score; DIET/NUTR=diet and nutritional biomarkers z-score variable (2 dietary quality measures and 4 nutritional biomarkers); HEALTH=Health-related factors as mean of z-scores for allostatic load, self-rated health, co-morbidity index and body mass index; N=Number of participants; PA=Physical activity z-score variable (3 measured variables); PCA=Principal Components Analysis; SES=Socio-economic status mean of z-scores composed of poverty income ratio and education (years); SMOKING=smoking z-score variable (2 measured variables); SS=Social Support z-score variable (5 measured variables). See Methods section for more details.

* P<0.05 for null hypothesis that β=0.

**APPENDIX III. COGNITIVE PERFORMANCE TESTS and PCA score**

*Word recall*

An interviewer-administered word recall test was given during the Household Adult Questionnaire. The interviewer listed the following words: “apple”, “table” and “penny” and asked the participant to repeat and remember the words. Up to three repetition trials were conducted until the participant named all three correctly. Participants were asked to remember all three words again after an intervening math test (serial 3’s) (see below). The measure of cognitive performance was the number of words correctly recalled (WR-CORR) as well as the number of trials to recall the words (WR-TRIALS) ^18, 19^. The former score was inverted by multiplying it by -1, to reflect poorer performance with higher score.

*Story recall*

An interviewer-administered story recall test was given during the examination in the MEC or during the home examination. The interviewer read a brief story to participants who were asked to recall details about the story. The story was:

“*Three children were alone at home and the house caught on fire. A brave fireman managed to climb in a back window and carry them to safety. Aside from minor cuts and bruises, all were well*.” From this story, six ideas were recorded as recounted or not and those were: “three children”, “house on fire”, “fireman climbed in”, “children rescued”, “minor injuries”, “everyone well”. The number of correctly recounted ideas (SR-CORR) was used to assess cognitive performance ^18, 19^. This score was inverted by multiplying it by -1, to reflect poorer performance with higher score.

*Math/arithmetic test (Serial 3’s)*

Participants were asked to subtract $3 from $20 and keep subtracting until asked to stop. The total number of incorrectly computed subtractions constituted a measure of poor cognitive performance (MATH-INC) ^18, 20^.

*Poor Cognitive Performance construct*

An exploratory factor analysis (EFA) was conducted on a random half sample of NHANES III participants with complete cognitive test scores. Test scores were coded to reflect poorer performance with higher score. The number of factors extracted was determined using the eigenvalue>1 rule. If more than one factor was extracted, varimax rotation was applied and factors were labelled based on a factor loading >0.40 cut-point. The PCA component(s) obtained reflecting poorer cognitive performance with higher score were used as the most proximate predictors to the main outcomes of interest in the generalized structural equations (GSEM) model. On the full sample, a principal components analysis was applied using the same number of factors extracted based on _the_ half sample, and varimax rotation if applicable. The z-score was predicted using the regression method on the entire eligible sample with available data (i.e. 60+y with HMO exclusion), after PCA. The results of the PCA and the regression coefficients for a model with predictors being each measured variable and outcome being the PCA score are presented in **Tables** **III.1**

**Table III. 1** PCA for cognitive performance test measured variables, NHANES III, 1988-1994, Age_base_≥60y (N=4,734)

|  |  | Component Loading |  |
| --- | --- | --- | --- |
| MATH-INC |  | 0.45 |  |
| WR-CORR (×-1) |  | 0.57 |  |
| WR-TRIALS |  | 0.49 |  |
| SR-CORR (×-1) |  | 0.47 |  |
| _Cons |  | __ |  |
| Eigenvalue |  | 1.93 |  |
| % var explained |  | 48.3% |  |

*Abbreviations*: COGN=Cognitive performance principal component variable (4 measured variables); PCA=Principal components analysis.

* P<0.05 for null hypothesis that β=0.

**APPENDIX IV. Summary of Table 1 findings**

Overall, participants were on average younger at examination within the middle and higher income groups compared with the lowest income (PIR≤130%), with a linear dose-response relationship. Moreover, the highest income group had a significantly lower proportion “rural” compared with the lowest income category. Most notably, race/ethnicity distribution differed markedly across income groups, whereby the proportion of Non-Hispanic Black participants were 7.4% and 2.9% in the middle and highest income group as opposed to 19% in the lowest income group, with a similar patten observed among Mexican-Americans. Although mean household size did not differ across income groups, the proportion married among participants increased with income in a linear fashion. Importantly, DIET, NUTR, PA, ALCOHOL were higher with increased income, while the reverse was true for HEALTH and COGN PCA (poor health and cognitive performance scores).

**References**

1. NHANES and CMS Linked Data Overview. <https://www.cdc.gov/nchs/tutorials/NHANES-CMS/Orientation/Overview/index.htm>, 2012, Accessed Date Accessed 2012 Accessed.

2. National Center for Health Statistics DL. Underlying and Multiple Cause of Death Codes. 2015.

3. McCullough ML, Feskanich D, Rimm EB, Giovannucci EL, Ascherio A, Variyam JN *et al.* Adherence to the Dietary Guidelines for Americans and risk of major chronic disease in men. *Am J Clin Nutr* 2000; **72**(5)**:** 1223-1231.

4. Beydoun MA, Fanelli-Kuczmarski MT, Allen A, Beydoun HA, Popkin BM, Evans MK *et al.* Monetary Value of Diet Is Associated with Dietary Quality and Nutrient Adequacy among Urban Adults, Differentially by Sex, Race and Poverty Status. *PLoS One* 2015; **10**(11)**:** e0140905.

5. Kuczmarski MF, Mason MA, Allegro D, Zonderman AB, Evans MK. Diet quality is inversely associated with C-reactive protein levels in urban, low-income African-American and white adults. *Journal of the Academy of Nutrition and Dietetics* 2013; **113**(12)**:** 1620-1631.

6. Raffensperger S, Kuczmarski MF, Hotchkiss L, Cotugna N, Evans MK, Zonderman AB. Effect of race and predictors of socioeconomic status on diet quality in the HANDLS Study sample. *Journal of the National Medical Association* 2010; **102**(10)**:** 923-930.

7. Seeman T, Merkin SS, Crimmins E, Koretz B, Charette S, Karlamangla A. Education, income and ethnic differences in cumulative biological risk profiles in a national sample of US adults: NHANES III (1988-1994). *Social science & medicine* 2008; **66**(1)**:** 72-87.

8. Gunter EW, Lewis, B. G., Koncikowski, S. M. . Laboratory Procedures used for the Third National Health and Nutrition Examination Survey (NHANES III), 1988–1994. In: US Department of Health and Human Services CfDCaP, Hyattsville, MD (ed).1996.

9. Fung TT, McCullough ML, Newby PK, Manson JE, Meigs JB, Rifai N *et al.* Diet-quality scores and plasma concentrations of markers of inflammation and endothelial dysfunction. *Am J Clin Nutr* 2005; **82**(1)**:** 163-173.

10. Visser M, Kritchevsky SB, Newman AB, Goodpaster BH, Tylavsky FA, Nevitt MC *et al.* Lower serum albumin concentration and change in muscle mass: the Health, Aging and Body Composition Study. *Am J Clin Nutr* 2005; **82**(3)**:** 531-537.

11. Ridker PM. Cardiology Patient Page. C-reactive protein: a simple test to help predict risk of heart attack and stroke. *Circulation* 2003; **108**(12)**:** e81-85.

12. Alberti KG, Zimmet PZ. Definition, diagnosis and classification of diabetes mellitus and its complications. Part 1: diagnosis and classification of diabetes mellitus provisional report of a WHO consultation. *Diabetic medicine : a journal of the British Diabetic Association* 1998; **15**(7)**:** 539-553.

13. Expert Panel on Detection E, Treatment of High Blood Cholesterol in A. Executive Summary of The Third Report of The National Cholesterol Education Program (NCEP) Expert Panel on Detection, Evaluation, And Treatment of High Blood Cholesterol In Adults (Adult Treatment Panel III). *Jama* 2001; **285**(19)**:** 2486-2497.

14. Golden S, Boulware LE, Berkenblit G, Brancati F, Chander G, Marinopoulos S *et al.* Use of glycated hemoglobin and microalbuminuria in the monitoring of diabetes mellitus. *Evidence report/technology assessment* 2003; (84)**:** 1-6.

15. Osei K, Rhinesmith S, Gaillard T, Schuster D. Is glycosylated hemoglobin A1c a surrogate for metabolic syndrome in nondiabetic, first-degree relatives of African-American patients with type 2 diabetes? *The Journal of clinical endocrinology and metabolism* 2003; **88**(10)**:** 4596-4601.

16. Seccareccia F, Pannozzo F, Dima F, Minoprio A, Menditto A, Lo Noce C *et al.* Heart rate as a predictor of mortality: the MATISS project. *American journal of public health* 2001; **91**(8)**:** 1258-1263.

17. Lenfant C, Chobanian AV, Jones DW, Roccella EJ, Joint National Committee on the Prevention DE, Treatment of High Blood P. Seventh report of the Joint National Committee on the Prevention, Detection, Evaluation, and Treatment of High Blood Pressure (JNC 7): resetting the hypertension sails. *Hypertension* 2003; **41**(6)**:** 1178-1179.

18. National Health and Nutrition Examination Survey III Household Adult data file documentation <ftp://ftp.cdc.gov/pub/Health_Statistics/NCHS/Datasets/NHANES/NHANESIII/2A/ADULTX-acc.pdf>. 1996, Accessed Date Accessed 1996 Accessed.

19. Krieg EF, Jr., Butler MA, Chang MH, Liu T, Yesupriya A, Dowling N *et al.* Lead and cognitive function in VDR genotypes in the third National Health and Nutrition Examination Survey. *Neurotoxicol Teratol* 2010; **32**(2)**:** 262-272.

20. Teng EL, Chui HC. The Modified Mini-Mental State (3MS) examination. *J Clin Psychiatry* 1987; **48**(8)**:** 314-318.

**TABLE S1.** Racial/ethnic disparities (Non-NHW vs. NHW) in incident all-cause dementia across income groups (≥60y, Unweighted N=4,570; Weighted N=35,439,687): Cox proportional hazards models; NHANES III, 1988-1994^a^

|  |  | **Overall** | | **Lowest income group**  **Poverty income ratio, PIR≤130%** | | **Middle income group**  **130%<PIR≤300%** | | **Highest income group**  **PIR>300%** | |  |
| --- | --- | --- | --- | --- | --- | --- | --- | --- | --- | --- |
| **Unweighted sample** |  | **N=4,570** | | **N=1,444** | | **N=1,817** | | **N=1,309** | |  |
|  |  | Log_e_(HR) | (SE) | Log_e_(HR) | (SE) | Log_e_(HR) | (SE) | Log_e_(HR) | (SE) |  |
|  |  |  |  |  |  |  |  |  |  |  |
| Model 1 |  |  |  |  |  |  |  |  |  |  |
| Non-White vs. NHW |  | +0.082 | (0.088) | -0.043 | (0.138) | -0.123 | (0.188) | -0.071 | (0.192) |  |
| Model 2 |  |  |  |  |  |  |  |  |  |  |
| Non-White vs. NHW |  | -0.068 | (0.095) | -0.205 | (0.179) | -0.178 | (0.197) | -0.084 | (0.187) |  |
| SES |  | **-0.216** | **(0.07)**** | -0.294 | (0.197) | **-0.381** | **(0.165)*** | +0.052 | (0.131) |  |
| Model 3 |  |  |  |  |  |  |  |  |  |  |
| Non-White vs. NHW |  | -0.086 | (0.098) | -0.176 | (0.180) | -0.183 | (0.200) | -0.117 | (0.187) |  |
| SES |  | **-0.146** | **(0.068)*** | -0.232 | (0.211) | -0.276 | (0.153) | +0.087 | (0.130) |  |
| *SMOKING* |  | *+0.100* | *(0.07)* | *+0.059* | *(0.112)* | *+0.100* | *(0.097)* | *+0.096* | *(0.102)* |  |
| *SS* |  | *-0.028* | *(0.08)* | ***-0.414*** | ***(0.160)**** | *+0.026* | *(0.123)* | *+0.127* | *(0.122)* |  |
| *NUTR* |  | *-0.015* | *(0.061)* | *-0.007* | *(0.098)* | *-0.078* | *(0.080)* | *+0.066* | *(0.126)* |  |
| *DIET* |  | ***-0.120*** | ***(0.048)**** | *+0.031* | *(0.093)* | ***-0.160*** | ***(0.071)**** | *-0.201* | *(0.095)** |  |
| *PA* |  | ***-0.238*** | ***(0.057)****** | *-0.218* | *(0.137)* | ***-0.264*** | ***(0.089)***** | *-0.186* | *(0.102)* |  |
| *ALCOHOL* |  | *-0.023* | *(0.041)* | *+0.027* | *(0.068)* | *-0.040* | *(0.061)* | *-0.008* | *(0.053)* |  |
| Model 4 |  |  |  |  |  |  |  |  |  |  |
| Non-White vs. NHW |  | -0.097 | (0.097) | -0.225 | (0.183) | -0.180 | (0.199) | -0.129 | (0.181) |  |
| SES |  | -0.136 | (0.068) | -0.236 | (0.201) | -0.261 | (0.157) | +0.091 | (0.129) |  |
| *SMOKING* |  | *+0.096* | *(0.060)* | *+0.063* | *(0.111)* | *+0.099* | *(0.097)* | *+0.093* | *(0.100)* |  |
| *SS* |  | *-0.025* | *(0.078)* | ***-0.400*** | ***(0.151)**** | *+0.029* | *(0.123)* | *+0.125* | *(0.124)* |  |
| *NUTR* |  | *-0.016* | *(0.061)* | *-0.016* | *(0.100)* | *-0.080* | *(0.081)* | *+0.068* | *(0.126)* |  |
| *DIET* |  | ***-0.115*** | ***(0.047)**** | *+0.046* | *(0.091)* | *-0.157* | *(0.072)* | ***-0.200*** | ***(0.096)**** |  |
| *PA* |  | ***-0.210*** | ***(0.059)***** | *-0.189* | *(0.137)* | *-0.241* | *(0.098)* | *-0.166* | *(0.104)* |  |
| *ALCOHOL* |  | *-0.018* | *(0.039)* | *+0.027* | *(0.068)* | *-0.035* | *(0.059)* | *-0.006* | *(0.052)* |  |
| *HEALTH* |  | ***+0.153*** | ***(0.074)**** | *+0.215* | *(0.125)* | *+0.097* | *(0.130)* | *+0.104* | *(0.087)* |  |
| Model 5 |  |  |  |  |  |  |  |  |  |  |
| Non-White vs. NHW |  | -0.190 | (0.095) | -0.272 | (0.187) | -0.258 | (0.200) | -0.182 | (0.194) |  |
| SES |  | -0.070 | (0.065) | -0.134 | (0.205) | -0.104 | (0.156) | +0.122 | (0.126) |  |
| *SMOKING* |  | *+0.098* | *(0.058)* | *+0.080* | *(0.108)* | *+0.087* | *(0.091)* | *+0.090* | *(0.099)* |  |
| *SS* |  | *-0.001* | *(0.081)* | ***-0.369*** | ***(0.153)**** | *+0.044* | *(0.126)* | *+0.143* | *(0.134)* |  |
| *NUTR* |  | *-0.011* | *(0.060)* | *-0.012* | *(0.099)* | *-0.064* | *(0.081)* | *+0.062* | *(0.130)* |  |
| *DIET* |  | ***-0.103*** | ***(0.046)**** | *+0.057* | *(0.091)* | ***-0.151*** | ***(0.069)**** | *-0.188* | *(0.100)* |  |
| *PA* |  | ***-0.186*** | ***(0.061)***** | *-0.170* | *(0.134)* | ***-0.226*** | ***(0.098)**** | *-0.139* | *(0.107)* |  |
| *ALCOHOL* |  | *-0.014* | *(0.038)* | *+0.023* | *(0.067)* | *-0.036* | *(0.058)* | *+0.006* | *(0.051)* |  |
| *HEALTH* |  | ***+0.150*** | ***(0.072)**** | *+0.207* | *(0.121)* | *+0.090* | *(0.130)* | *+0.115* | *(0.087)* |  |
| *COGN* |  | ***+0.182*** | ***(0.025)****** | ***+0.119*** | ***(0.048)**** | ***+0.243*** | ***(0.043)****** | ***+0.199*** | ***(0.072)****** |  |
|  |  |  |  |  |  |  |  |  |  |  |

*Abbreviations*: ALCOHOL= alcohol consumption, z-score; COGN=Cognitive performance principal component variable (4 measured variables); DIET/NUTR=diet and nutritional biomarkers z-score variable (2 dietary quality measures and 4 nutritional biomarkers); HEALTH=Health-related factors as mean of z-scores for allostatic load, self-rated health, co-morbidity index and body mass index; HR=Hazard Ratio; LIFESTYLE=Lifestyle-related factors composed of social support, physical activity, diet/nutritional biomarkers, smoking and alcohol consumption using means of z-scores for related measured variables; MA=Mexican American; N=Number of participants; N’=number of observations; NHANES III=Third National Health and Nutrition and Examination Survey; NHB=Non-Hispanic Blacks; NHW=Non-Hispanic White; PA=Physical activity z-score variable (3 measured variables); RACE_ETHN=racial/ethnic contrast; SES=Socio-economic status mean of z-scores composed of poverty income ratio and education (years); SMOKING=smoking z-score variable (2 measured variables); SS=Social Support z-score variable (5 measured variables). See Methods section for more details.

^a^ Values are β ± SE (Log_e_(HR)), considering sampling design complexity (PSU and strata), across 5 imputations with 10 iterations.

Model 1: adjusted for age and sex; Model 2: adjusted for demographic factors other than age and SES score; Model 3: Model 2 further adjusted for lifestyle-related factors (average of z-scores of measured variables for SMOKING, ALCOHOL, DIET, NUTR, SS and PA); Model 4: Model 3 + health-related factors (HEALTH score); Model 5: Full model with cognitive test PCA score.

^b^ P<0.05 for POVSTAT×RACE_ETHN interaction in unstratified model; ^c^ P<0.05 for POVSTAT×SES interaction in unstratified model.

*P<0.05 **P<0.01 ***P<0.001 for null hypothesis of Log_e_(HR)=0.

**SUPPLEMENTARY FIGURE LEGEND**

**FIGURE S1**. **Participant Flowchart**

*Abbreviations*: AD=Alzheimer’s Disease; CMS=Centers for Medicare and Medicaid; NHANES=National Health and Nutrition Examination Surveys

**FIGURE S2**. **GSEM full model and hypothesized pathway**

*Abbreviations*: AD=Alzheimer’s Disease; ALCOHOL= alcohol consumption, z-score; COGN=Cognitive performance principal component variable (4 measured variables); DIET/NUTR=diet and nutritional biomarkers z-score variable (2 dietary quality measures and 4 nutritional biomarkers); HEALTH=Health-related factors as mean of z-scores for allostatic load, self-rated health, co-morbidity index and body mass index; LIFESTYLE=Lifestyle-related factors composed of social support, physical activity, diet/nutritional biomarkers, smoking and alcohol consumption using means of z-scores for related measured variables; N’=number of observations; MA=Mexican American; NHANES III=Third National Health and Nutrition and Examination Survey; NHB=Non-Hispanic Blacks; NHW=Non-Hispanic White; PA=Physical activity z-score variable (3 measured variables); RACE_ETHN=racial/ethnic contrast; SES=Socio-economic status mean of z-scores composed of poverty income ratio and education (years); SMOKING=smoking z-score variable (2 measured variables); SS=Social Support z-score variable (5 measured variables). See Methods section for more details.

**Notes: Plain arrows are statistically significant associations (p<0.05) within the hypothesized pathway; Dashed arrows are statistically significant associations (p<0.05) outside the hypothesized pathway.**
