# Supplementary material for: Pathways explaining racial/ethnic and socio-economic disparities in incident all-cause dementia among older US adults across income groups
Source: Transl Psychiatry. 2022 Nov 15;12:478. doi: 10.1038/s41398-022-02243-y (PMC9666623; doi:10.1038/s41398-022-02243-y)
Supplement: Supplementary file 2 — Figure S1 [file 41398_2022_2243_MOESM2_ESM.pptx]

## Slide 1
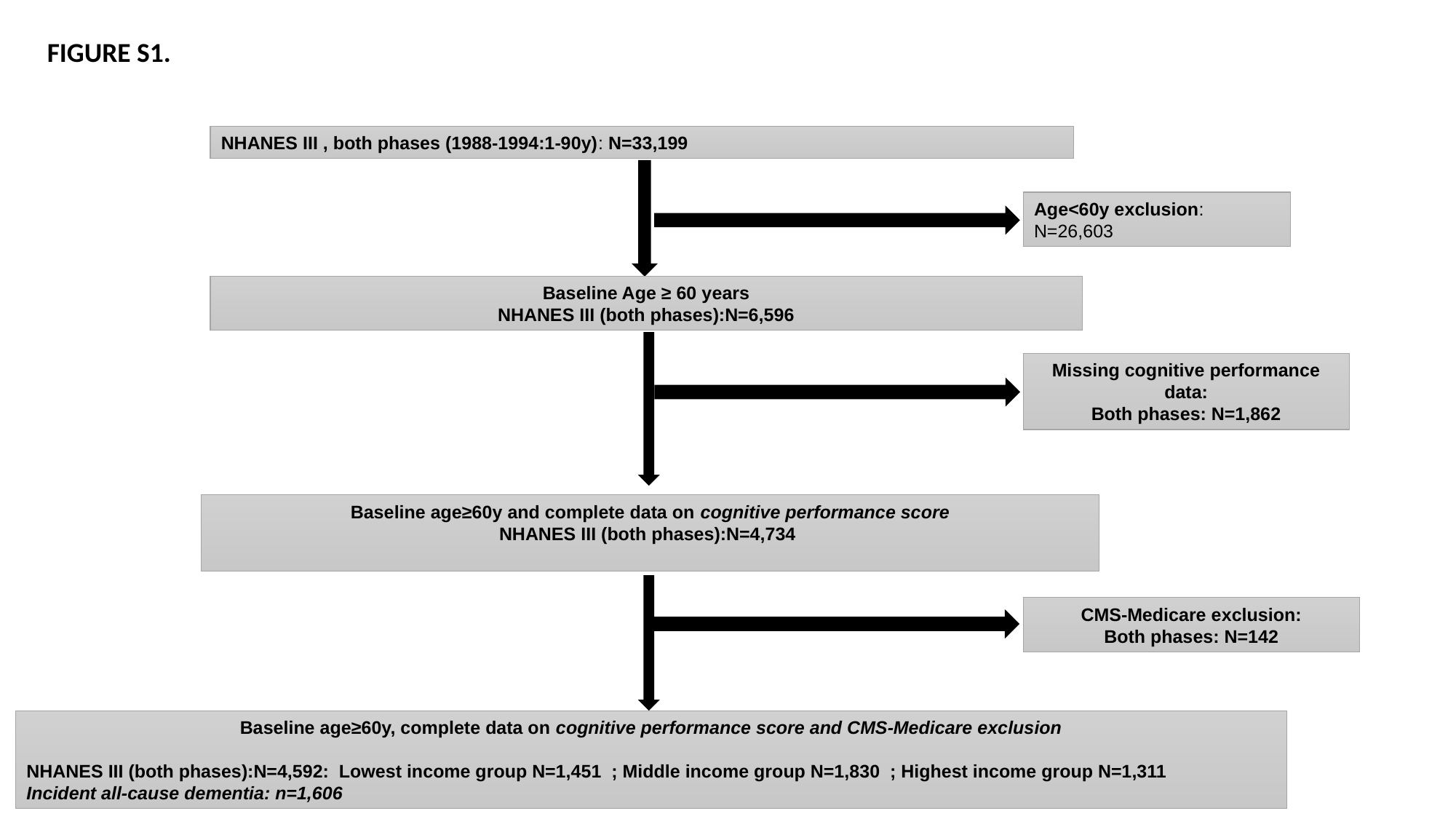

FIGURE S1.
NHANES III , both phases (1988-1994:1-90y): N=33,199
Age<60y exclusion:
N=26,603
Baseline Age ≥ 60 years
NHANES III (both phases):N=6,596
Missing cognitive performance data:
Both phases: N=1,862
Baseline age≥60y and complete data on cognitive performance score
NHANES III (both phases):N=4,734
CMS-Medicare exclusion:
Both phases: N=142
Baseline age≥60y, complete data on cognitive performance score and CMS-Medicare exclusion
NHANES III (both phases):N=4,592: Lowest income group N=1,451 ; Middle income group N=1,830 ; Highest income group N=1,311
Incident all-cause dementia: n=1,606
