# Supplementary figures and images for: Pathways explaining racial/ethnic and socio-economic disparities in incident all-cause dementia among older US adults across income groups
Source: Transl Psychiatry. 2022 Nov 15;12:478. doi: 10.1038/s41398-022-02243-y (PMC9666623; doi:10.1038/s41398-022-02243-y)

## Slide 1
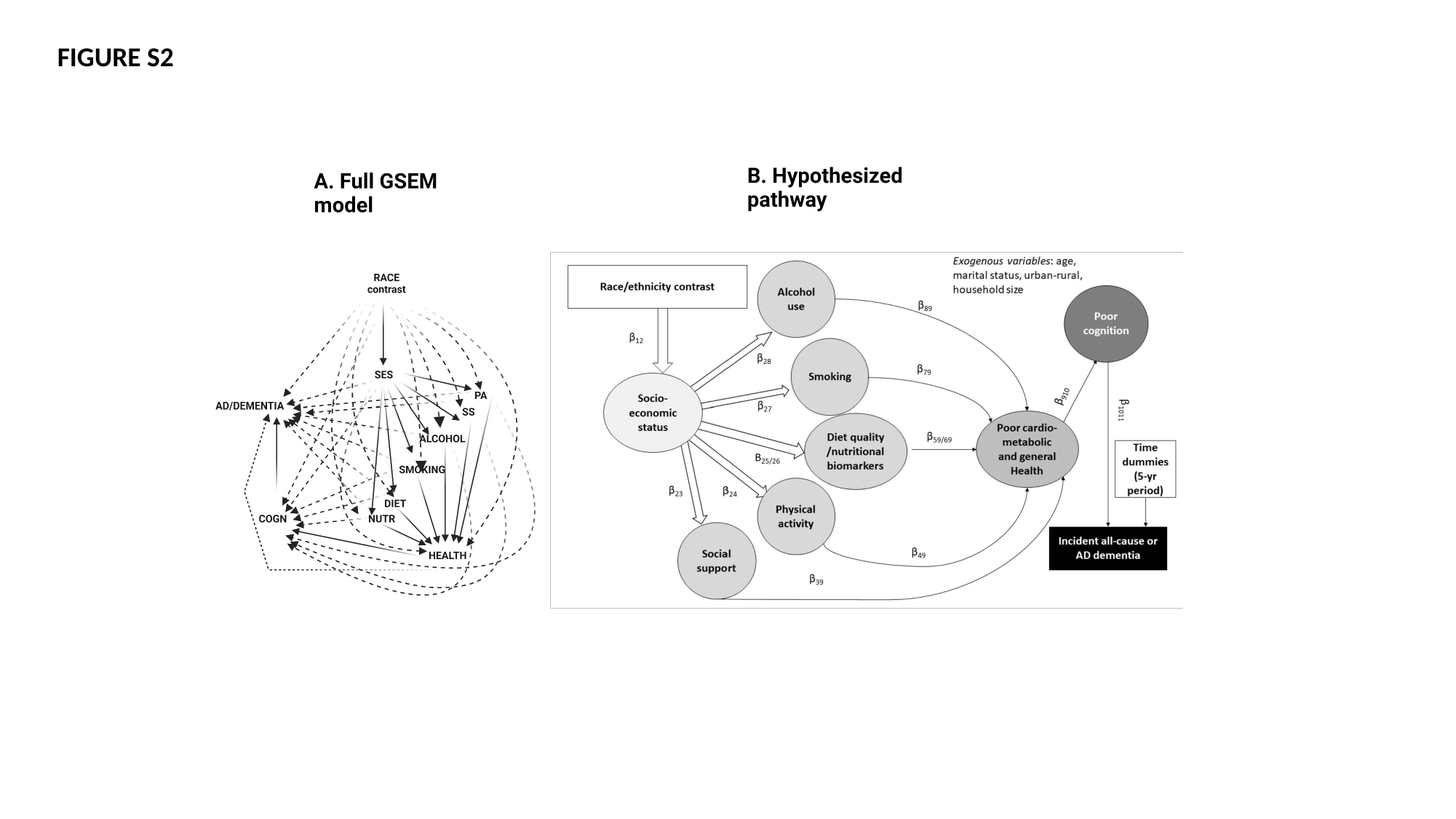

FIGURE S2

Supplement: Supplementary file 3 — Figure S2 [file 41398_2022_2243_MOESM3_ESM.pptx]
